# Genome-Wide Homozygosity Mapping Reveals Genes Associated With Cognitive Ability in Children From Saudi Arabia

**DOI:** 10.3389/fgene.2019.00888

**Published:** 2019-09-18

**Authors:** Sergey A. Kornilov, Mei Tan, Abdullah Aljughaiman, Oxana Yu Naumova, Elena L. Grigorenko

**Affiliations:** ^1^Baylor College of Medicine, Department of Molecular and Human Genetics, Houston, TX, United States; ^2^Department of Psychology, University of Houston, Houston, TX, USA; ^3^Special Education Department, King Faisal University, Ahsaa, Saudi Arabia; ^4^Vavilov Institute of General Genetics, Russian Academy of Sciences, Moscow, Russia; ^5^Child Study Center, Yale University, New Haven, CT, USA; ^6^Moscow State University for Psychology and Education, Moscow, Russia

**Keywords:** intelligence, cognitive ability, genome-wide association study, homozygosity mapping, consanguinity, *GRIA4*

## Abstract

Recent studies of the genetic foundations of cognitive ability rely on large samples (in extreme, hundreds of thousands) of individuals from relatively outbred populations of mostly European ancestry. Hypothesizing that the genetic foundation of cognitive ability depends on the broader population-specific genetic context, we performed a genome-wide association study and homozygosity mapping of cognitive ability estimates obtained through latent variable modeling in a sample of 354 children from a consanguineous population of Saudi Arabia. Approximately half of the sample demonstrated significantly elevated homozygosity levels indicative of inbreeding, and among those with elevated levels, homozygosity was negatively associated with cognitive ability. Further homozygosity mapping identified a specific run, inclusive of the *GRIA4* gene, that survived corrections for multiple testing for association with cognitive ability. The results suggest that in a consanguineous population, a notable proportion of the variance in cognitive ability in the normal range in children might be regulated by population-specific mechanisms such as patterns of elevated homozygosity. This observation has implications for the field’s understanding of the etiological bases of intelligence and its variability around the world.

## Introduction

Studies of the genetic foundations of cognitive ability published in the last decade have made significant progress in elucidating the complex etiology of cognitive ability as a human trait that is influenced by a multitude of genetic factors. Two recent meta-analyses are particularly noteworthy, as they signify a leap in sample size, to n = 78,308 (Sniekers et al., 2017) and n = 300,486 (Davies et al., 2018), which may generate enough statistical power to detect small-effect-size genetic loci that constitute some of these factors. The former (Sniekers et al., 2017) revealed 18 independent genome-wide significant loci associated with 336 single nucleotide polymorphisms (SNPs), with the majority being previously unreported, although many of the newly implicated genes (e.g., *MEF2C* and *SHANK3*) are known for their functions in neural development. The latter (Davies et al., 2018) is the largest meta-analysis of cognitive ability to date (combining the CHARGE and COGENT consortia and the UK Biobank data); it implicated 148, including 58 newly reported, loci. The derived polygenic scores predicted up to 4.31% of the variance in cognitive ability, and several pathways, including the positive regulation of nervous system development, were overrepresented among the genes highlighted by this meta-genome-wide association study (GWAS).

These findings are supported by results from recent large-scale GWAS studies of educational attainment that indicate that this proxy phenotype for cognitive ability is also governed by substantial genetic control. Genome-wide significant findings in the studied cohorts have identified biologically plausible pathways for cognitive ability [e.g., among the 74 loci identified, *MEF2C* was also significantly associated with educational attainment (Okbay et al., 2016)], and derived polygenic scores explained up to 2% of the variance in the trait (Rietveld et al., 2013). Polygenic scores for educational attainment also predicted cognitive behavioral phenotypes across the lifespan in another longitudinal study (Belsky et al., 2016).

This steady stream of findings implicating multiple small-effect loci as contributors to the genetic control of cognitive ability puts emphasis on the substantial statistical power required for their reliable detection under the common variant hypothesis. Notably, around 50% of the estimated chip heritability [i.e., the proportion of variance in ability estimates cumulatively explained by the modeled genetic distances between individuals using a large number of genotyped markers (Plomin and Von Stumm, 2018)] can be attributed to variants located in genomic regions that are under negative selection (Hill et al., 2016). Concordantly, rare functional coding alleles, when associated with cognitive ability, are more likely to be detrimental rather than beneficial (Spain et al., 2016) to it.

The large-scale studies mentioned earlier were performed in samples drawn from populations of European ancestry, characterized by relatively low levels of homozygosity due to the low prevalence of consanguinity. Much less used are samples with genetic backgrounds characterized by excessive homozygosity. Given that cryptic parental relatedness and consanguinity have been found to be significantly negatively associated with cognitive ability (Howrigan et al., 2016), as well as severity of syndromic intellectual disability (Gandin et al., 2015) even in relatively outbred individuals, homozygosity can be considered an additional window into the genetic regulation of intelligence. This hypothesis is supported by the presence of significant negative associations between SNP-based inbreeding coefficients and a range of traits (including educational attainment) in a sample of approximately 5,500 unrelated Finnish individuals (Verweij et al., 2014) and, complementarily, the absence of association between runs of homozygosity (ROH) burden and cognitive ability in a UK sample of n = 2,329 unrelated individuals (Power et al., 2014b).

More recent and larger studies of cohorts where elevated autozygosity is not observed provide further evidence for the role of homozygosity in cognitive ability. In a well-powered study of the effects of homozygosity on 16 health-related traits in a sample of n = 354,224 individuals, elevated homozygosity was associated with decreased cognitive ability across study cohorts (Joshi et al., 2015). Similarly, autosomal homozygosity burden was associated with lower general intelligence in the UK Biobank sample of almost 400,000 individuals (Johnson et al., 2018). Thus, the particular constellation of factors involved in the genetic regulation of cognitive ability might depend on a complex polygenic genomic background, that is, population-specific mechanisms such as increased homozygosity (i.e., the presence of long stretches of homozygous DNA reflecting parental relatedness that may have deleterious effects on gene function in a recessive fashion) potentially account for additional variance in cognitive ability that has not been captured by individual and even gene-based analyses in populations not enriched for homozygosity-related haplotypic variation.

The goal of this study was to examine patterns in the genetic foundation of cognitive ability in a sample of children from the Kingdom of Saudi Arabia (KSA), a country that historically has had a significant [i.e., up to 57.7% prevalence in one study (El-Hazmi et al., 1995)] and stable (Warsy et al., 2014) number of consanguineous marriages. The substantial negative effects of consanguinity and homozygosity on Mendelian traits and the elevated prevalence of rare recessive diseases and disorders are well-established in the Gulf countries, including in recent reports from Saudi Arabia (Alfares et al., 2017; Majid et al., 2017) and Qatar (Bener and Mohammad, 2017). To date, however, no study has directly examined genome-wide homozygosity patterns with respect to their contribution to cognitive ability in Middle Eastern countries (for a review of published studies, see Ceballos et al., 2018b), despite the rising awareness of the possible contribution of consanguinity-driven homozygosity not only to rare Mendelian disorders and intellectual disability in consanguineous populations but also to the etiology of complex traits and common disorders (Erzurumluoglu et al., 2016).

Given the recent findings from exome-sequencing studies in Saudi Arabia that documented a high diagnostic yield of variants associated with a plethora of disorders (Alfares et al., 2017; Monies et al., 2017), among them intellectual disability, we hypothesized that individual differences in cognitive ability in this population might be partly regulated by regions of homozygosity. We also hypothesized that these regions would be placed in-between common and rare variants on the continuum of effect sizes and that elevated levels of homozygosity (and allelic ROHs) would increase our statistical power to detect such effects even in a sample of modest size. This hypothesis is contextualized by 1) observations that long ROHs are enriched for deleterious variation that amplify the effects of mildly deleterious variants that exist in a homozygous state (Szpiech et al., 2013) and 2) recent successes in the homozygosity mapping of neurodevelopmental attention-deficit/hyperactivity disorder in a small sample of Saudi siblings with attention-deficit/hyperactivity disorder, which identified several new candidates for the disorder in a sample of limited size (Shinwari et al., 2017). Therefore, by capitalizing on the unique nature of the sample from the consanguineous population of KSA, recent advances in model-based identification of long tracts of homozygosity (Ceballos et al., 2018a; Ceballos et al., 2018b), and quantification of latent cognitive ability trait estimates via structural equation modeling, we sought to perform a GWAS and homozygosity mapping of cognitive ability in a sample of children from Saudi Arabia using a genotyping platform with increased exonic coverage, enabling the identification of long genic ROHs.

## Materials and Methods

### Participants

Participants for the genetic study were recruited from a larger sample enrolled in an epidemiological study of cognitive ability in children in Saudi Arabia. For this epidemiological study, a total of n = 7,186 children in the age range from 7.34 to 18.71 years (*M* = 12.28, *SD* = 1.81; 4,682 males and 2,504 females) were recruited from local schools in seven major regions of Saudi Arabia: Abha (n = 605), Khamis-Mushyat (n = 585), Tabuk (n = 1,095), Al-Jubail (n = 1,004), Jeddah (n = 2,272), and Al-Hassa (n = 1,625).

Using the data from the epidemiological study, n = 942 children of Saudi Arabian ancestry were identified for the genetic study by the school officials based on their performance on the cognitive ability battery administered as part of the epidemiological study. Proband selection was performed separately for each grade using the 85^th^ percentile cut-off across the sum of all administered items, and the genetic study sample was therefore expected to be enriched for high general cognitive ability. Thus, although the data reported in this manuscript are based on the sample of children selected to represent the high tail of the cognitive ability distribution, the final cognitive ability estimates used for the genetic association were obtained after a careful examination of the psychometric properties of Aurora and appropriate revisions (i.e., exclusion of misfitting items, see discussion later). Thus, the resulting scores covered a broad range of the distribution in general intelligence, albeit with a notable shift toward higher scores (see **Supplementary Material**).

Saliva was collected from every consenting proband (n = 354) in the age range from 8.23 to 13.93 (*M* = 11.33, *SD* = 1.36; 273 or 77% were boys). These children comprised the main sample of the study reported here. All children and their parents were born in KSA to Saudi Arabian parents according to a self-report (see also **Supplementary Table S1** for a more detailed study sample demographics breakdown).

The study protocol and procedures were approved by the Internal Review Boards of Yale University, University of Houston, and King Faisal University (Saudi Arabia). Parents of children participating in the study and authorities at the appropriate educational institutes provided written informed consent in accordance with the Declaration of Helsinki.

### Cognitive Ability Assessment

All children were administered a paper-and-pencil cognitive ability assessment in a group format in a controlled school environment. Specifically, we used the Aurora (Chart et al., 2009; Tan et al., 2009; Hein et al., 2015) assessment to obtain estimates of cognitive ability in the large sample of n = 7,186 children to adequately model the distributions of population parameters. The version of the instrument used for this study was developed taking into account relevant cultural and linguistic factors (Tan et al., 2009) across a number of cultures, countries, and languages. In the absence of convergent or predictive (with respect to, for example, academic outcomes) validity data on the instrument in Saudi Arabia, we performed a careful psychometric analysis and construct validation of the instrument as reported later (more information is available from the authors upon request).

We used the data from the “general” cognitive ability module of Aurora that was designed to capture variability in children’s general cognitive ability and appropriately model domain-specific variation. The test battery includes nine subtests designed to fill a 3 (domain: verbal, numerical, and figural) × 3 (task: classification, series, and analogies) test design matrix, with the number of items ranging from 8 to 22 per subtest (8 to 18 after item exclusion, see later). The order of the Aurora subtests within the administered testlets was randomized across data collection sites. Children were allowed 45 min to complete the assessment. Test administration was governed by trained assessors. If the child did not attempt a subtest, the data for that subtest were coded as missing.

The calculation of latent ability trait estimates was performed using a two-step approach. First, patterns of responses to test items were scored using item response theory. Specifically, estimates of subtest-level abilities were obtained by fitting a set of 2-parametric models to the binary response vectors as implemented in the R *mirt* (Chalmers, 2012) library, thereby estimating both item difficulty and item discrimination. Local fit indices and item characteristic curves were analyzed, and items were removed from the dataset when local misfit was diagnosed, and when removal resulted in the increase of variance in item response patterns explained by the item response theory model (see also **Supplementary Table S2** for the description of subtest-level parameters).

**Figure 1:**
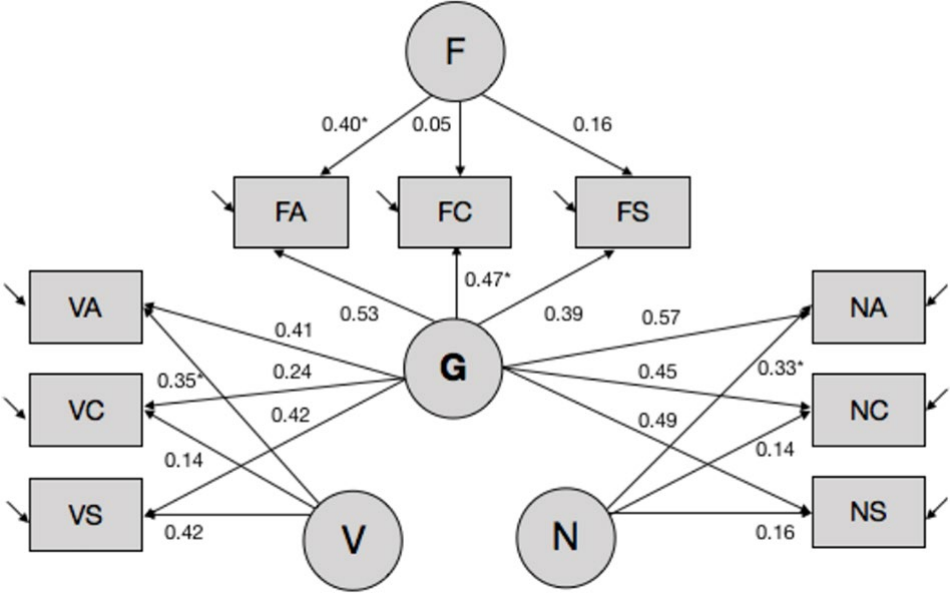
Latent variable model of Aurora. *(s) denote coefficients that were fixed to 1 to enable model identification and parameter estimation. Other parameters were estimated freely. Covariances between latent variables were fixed to 0. Aurora-G, general cognitive ability; Aurora-V, verbal cognitive ability; Aurora-N, numerical cognitive ability; Aurora-F, figural (spatial) cognitive ability. Subtests: VA, verbal analogies; VC, verbal classification; VS, verbal series; NA, numerical analogies; NC, numerical classification; NS, numerical series; FA, figural analogies; FC, figural classification; FS, figural series.

Second, we performed latent variable modeling of subtest-level ability estimates by fitting a set of alternative models to the data using EQS 6.1. software (Bentler, 2006). The models differed in the number of estimated latent trait variances and covariance, and corresponding pattern and error loadings. Overall, several models displayed good fit to the data, and the bifactor model (see **Figure 1**, also **Table S3**) was chosen as the final model based on theoretical considerations as well as recent data documenting the superior fit of the bifactor model to the data in most cases (Cucina and Byle, 2017). Briefly, the bifactor models postulates the existence of orthogonal specific ability factors that load on their respective items [i.e., in this case—domain-specific factors of verbal (Aurora-V), numerical (Aurora-N), and spatial/figural ability (Aurora-F)], while all items load on the common general factor (Aurora-G). Missing data were modeled using the robust pairwise covariance approach, and latent ability estimates were computed using the generalized least squares estimator.

Estimates of latent ability traits were adjusted for gender, age, and its quadratic term age^2^ and the interaction between age and gender terms using the robust implementation of linear mixed modeling in the *robustlmm* (Koller, 2016) package for *R*. The models were fitted separately for each phenotype (Aurora-V, Aurora-N, Aurora-F, and Aurora-G) and included a school ID variable as a random effect to account for the nested structure of the data obtained for the total sample of children, thereby increasing the precision of the parameter estimates.

### DNA Sample Collection, Processing, and Genotyping

All participating individuals provided a saliva sample using Oragene^TM^ OG-250 (DNA Genotek, Inc) self-collection kits. Saliva samples were processed at Yale University. DNA was extracted using the manufacturer’s protocol; DNA integrity was verified by agarose gel electrophoresis, and DNA concentration was determined with the Quant-iT dsDNA High-Sensitivity Assay Kit using a Qubit 2.0 fluorometer (ThermoFisher Scientific, Inc). Genotyping was performed by Illumina, Inc using the FastTrack genotyping service. To minimize batch effects, the placement of samples on individual plates was block-randomized with respect to geographical region/school, gender, age, and cognitive ability (using the median-split on Aurora-G scores).

Samples were genotyped using the HumanCoreExome v1.2 BeadChip microarray that included a total of 550k SNP markers. The HumanCoreExome array is specifically enriched for exonic SNPs and low minor allele frequency (MAF) SNPs. Allele calling was performed using Illumina’s GenomeStudio. Individual SNPs were mapped onto the hg19 build; clustering results were examined visually, and clustering was adjusted manually when necessary.

All samples had a call rate > 95% and passed the reported vs. inferred gender check. Marker-based quality control included: removal of Illumina’s insertion/deletion markers (k = 9,497), removal of markers with call rate < 95% (k = 2,330), and removal of markers with MAF < 1% (k = 229,664). Thus, the resulting dataset included k = 285,340 autosomal markers, with an estimated density of 1 SNP per 9.965 kb.

Genetic principal components using the additive genotypic model were estimated after the linkage disequilibrium pruning of the dataset (with *r*^2^ threshold of 0.50, and a window size of 50; k = 171,399 markers) using SNP & Variation Suite (GoldenHelix, Inc).

### Homozygosity Mapping

Homozygosity mapping was performed using GARLIC (Szpiech et al., 2017). GARLIC detects ROH using a model-based approach and has an advantage over SNP counting methods with respect to the modeling of population-specific parameters, genotyping error rates, and microarray platform SNP density. Peri-centromeric and telomeric regions were excluded from the analyses using hg19 coordinates. The technical replicate-based genotyping error rate of 0.0019681673 was provided to the software with the *–weighted* flag enabled, and panel density-adjusted window size was automatically set to 62 SNPs.

In contrast to genotype counting methods such as implemented in PLINK, GARLIC utilizes the logarithm of odds score measure of homozygosity using a sliding window framework to infer ROHs, with distribution of logarithm of odds scores estimated through the use of a Gaussian kernel density estimator (KDE). KDE was computed using the data from all samples (i.e., KDE thinning was disabled). GARLIC uses a three-component mixture model to classify ROH calls into three categories. Sample ROHs were classified into Short (A; 75.43% of all calls, with an average length of 625 kb) ROHs reflecting homozygosity for ancestral haplotypes; Medium (B; 16.64%; length > 1.3 Mb; average length 1.9 Mb) ROHs; and Long (C; 7.93%, length > 4.48 Mb; average length 11.2 Mb) ROHs, reflecting recent population processes and consanguinity. ROH-driven homozygosity (f_ROH_) values were estimated separately for each class of ROHs and cumulatively, representing the percent of the mappable autosomal genome covered by ROHs (i.e., proportion out of 9,965 × 285,340 bp). A SNP-based homozygosity index f_SNP_ was calculated for each sample using the pruned dataset in SNP & Variation Suite.

### Copy Number Variant Calling

Copy number analysis was performed on 353 out of 354 samples after the exclusion of one sample with outlying *SD* of logR intensity values. To ensure the comparability of ROH and copy number variant (CNV) calls, probe intensity values, genotypes, and corresponding B allele frequencies were exported for k = 285,340 autosomal polymorphic markers that passed SNP-based quality control for the total sample. Probe intensity values were adjusted for genomic waves using the CNV quality control module of SNP & Variation Suite. Genomic wave-adjusted values were then winsorized using the *copynumber* (Nilsen et al., 2012) library for *R*, and missing values for no-call genotypes were imputed using the piecewise constant segmentation approach (pcf) implemented in *copynumber*.

We then used PennCNV (Wang et al., 2007) to perform CNV segmentation and calling. PennCNV uses Hidden Markov Model approach for evaluating copy number states in genome-wide microarray genotyping data. Analyses were performed with the following settings: CNV segments were required to have a minimum length of 10 SNPs and 100 kb; median-adjustment of BAF and probe intensity values was enabled. The population frequency of the B allele (*.pfb) and the Hidden Markov model file, with the expected intensity values for different copy number states for the HumanCoreExome panel that we used in the study, was downloaded from the PennCNV website (http://penncnv.openbioinformatics.org). Marker locations were verified against Illumina’s manifest files.

A total of k = 1,673 CNVs ranging in size from 10 kb to 1.3 Mb (*M* = 170 kb, median = 123 kb, SD = 163 kb) were called in the 353 analyzed samples, with 476 or 28% being copy number gain and the remaining 1,197 or 72% being copy number loss events.

### Statistical Analyses

A GWAS was performed using a linear mixed modeling approach implemented in GEMMA (Zhou and Stephens, 2012) using a standardized pairwise identity-by-state relatedness matrix to account for population structure and relatedness. P-values were estimated using the Wald method, and the threshold for genome-wide significance was estimated using the M_eff_ approach as implemented in simpleM (Gao et al., 2010) and multiplied by the number of tested phenotypes, with the resulting threshold value of *P* = 5.29 × 10^-8^ for four Aurora phenotypes (Aurora-V, Aurora-N, Aurora-F, and Aurora-G) combined.

Gene-based association analyses were carried out with nominal P-values from the GEMMA analysis using the effective chi-square gene-based test as implemented in KGG v4 (Li et al., 2010). Analyses were limited to protein-coding genes, and gene boundaries were padded with 10-kb regions for SNP assignment. P-values were combined for the four phenotypes and adjusted for multiple comparisons jointly using the Benjamini–Hochberg correction (Benjamini and Hochberg, 1995). P-values were combined for the four phenotypes and adjusted for multiple comparisons jointly using the Benjamini–Hochberg (Benjamini and Hochberg, 1995) correction. ROH and CNV association analyses were performed using the linear model, *lm()*, function in R, while controlling for the top five genetic principal components and f_SNP_ homozygosity. P-values were adjusted using the Benjamini–Hochberg correction. Latent class analysis (LCA) of homozygosity was performed using the R *mclust* (Scrucca et al., 2016) library. The goal of the LCA as an unsupervised data mining technique is to perform a data dimensionality reduction by identifying latent clusters of individuals in the multidimensional space of indicator variables. LCA, also known as finite mixture modeling, is a person-centered (as opposed to variable-centered) analytical framework that models the classification of individuals into unobserved subpopulations (latent classes) via the joint modeling of observed distributions as representing mixtures of distributions of two or more subpopulations (Vermunt and Madigson, 2002). We used LCA in the current study to explore CNV and ROH burden values to quantify the broad genomic background, aiming to identify homogenous subgroups of children with lower and higher levels of CNV and ROH burdens.

Since the LCA results suggested the presence of several latent clusters of individuals that differed in size, we used complementary methods to evaluate the contribution of f_ROH_ and CNV burdens to cognitive ability. For the smallest classes, nonparametric linear regression with permutation testing was performed with the *lmPerm()* package. ROH and CNV burden analyses in larger classes of individuals were conducted using quantile regression as implemented in the *quantreg* library for R. We chose to utilize this semi-parametric method because we hypothesized that the contribution of f_ROH_ might be uneven at different levels of cognitive ability. Therefore, the 25^th^, 50^th^, and 75^th^ quantiles were chosen for the analyses aimed at examining the contribution of homozygosity and structural variant burden at conditional low, moderate, and high levels of cognitive ability. Note that, in the current sample, these quantiles corresponded to average-high, high, and very high levels of cognitive ability (relative to the larger normative sample of children). Standard errors and P-values were obtained using bootstrapping, with 10,000 bootstrap samples. R-based analyses were performed using the Microsoft OpenMP implementation of R (http://mran.microsoft.com/) in the R Studio Server IDE environment, installed on a Linux bioinformatics high-performance computing workstation. Additional information regarding specific procedures used for CNV association analyses, analyses of relatedness using patterns of identity by descent-sharing, and ancestral composition of the sample is provided in the **Supplementary Material**.

## Results

### Single Nucleotide Polymorphism-Based Genome-Wide Association Study of Aurora-G Scores

**Figure 2:**
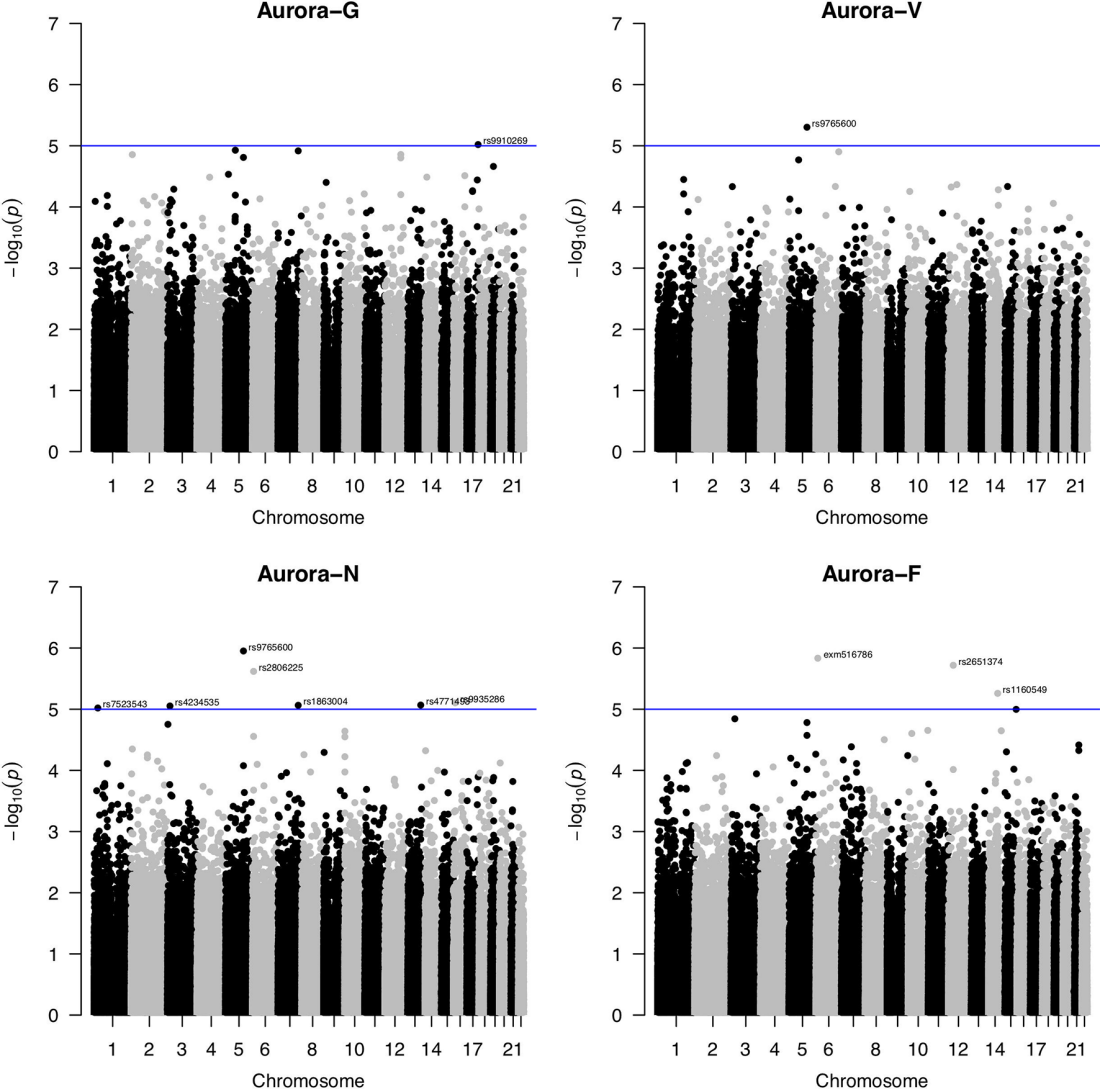
Manhattan plots for genome-wide association study of Aurora scores. Blue line indicates the nominal significance threshold of *P* = 1x10^-5^. Aurora-G, general cognitive ability; Aurora-V, verbal cognitive ability; Aurora-N, numerical cognitive ability; Aurora-F, figural/spatial cognitive ability.

SNP-based association analyses conducted using linear mixed modeling in GEMMA did not reveal any markers that would survive corrections for multiple testing (**Figure 2**). Using a suggestive association threshold of *P* = 1 × 10^-5^, we established a set of tentative association signals for 11 SNPs (summarized in **Table 1**). Several of these findings are noteworthy and will be briefly discussed here. First, a rare missense SNP, rs79027391, located in exon 1 of the human immunodeficiency virus type I enhancer binding protein 1 (*HIVEP1*; 6p24.1) gene, was strongly negatively associated with figural/spatial ability (Aurora-F). *HIVEP1* is a transcription factor that belongs to the ZAS family of proteins that bind to enhancer elements of viral as well as cellular genes. Mutations in a closely functionally related gene, *HIVEP2*, were recently shown to be associated with intellectual disability and developmental delay (Steinfeld et al., 2016).

**Table 1:** Suggestive SNP association signals for Aurora.

SNP	Chr	Position (bp)	Min/Maj	MAF	*B*	*SE*	*P*	Phenotype	Gene
rs7523543	1	25,186,716	G/A	0.37	-5.19	1.15	9.52 × 10^-6^	Aurora-N	*LOC105376876* (intron)
rs4234535	3	16,162,533	A/G	0.28	-5.24	1.16	8.84 × 10^-6^	Aurora-N	*GALNT15* (upstream of)
rs9765600	5	119,919,047	A/C	0.40	-5.18	1.04	1.12 × 10^-6^	Aurora-N	*PRR16* (intron)
					-4.64	1.00	4.97 × 10^-6^	Aurora-V	
rs79027391	6	12,122,102	G/T	0.02	-20.01	4.08	1.46 × 10^-6^	Aurora-F	*HIVEP1* (missense)
rs2806225	6	7,579,412	T/C	0.24	6.16	1.28	2.41 × 10^-6^	Aurora-N	*DSP* (intron)
rs1863004	7	135,053,638	G/A	0.24	-5.73	1.27	8.60 × 10^-6^	Aurora-N	*CNOT4* (intron)
rs2651374	12	32,582,858	C/T	0.46	-5.10	1.05	1.91 × 10^-6^	Aurora-F	*FGD4* (intron)
rs4771493	13	105,927,432	C/T	0.32	5.24	1.16	8.57 × 10^-6^	Aurora-N	*LOC105370344* (upstream of)
rs1160549	14	80,464,509	C/T	0.13	6.88	1.49	5.49 × 10^-6^	Aurora-F	*NRXN3* (downstream from)
rs9935286	16	12,594,359	C/T	0.22	5.97	1.32	7.86 × 10^-6^	Aurora-N	*SNX29* (intron)
rs9910269	17	75,049,645	T/C	0.24	-5.18	1.15	9.53 × 10^-6^	Aurora-G	*SEC14L1* (upstream of)

Min, minor allele; Maj, major allele; MAF, minor allele frequency; B, effect size estimate; SE, standard error; P, Wald p value (uncorrected); Phenotypes: Aurora-G, general cognitive ability; Aurora-V, verbal cognitive ability; Aurora-N, numerical cognitive ability; Aurora-F, figural cognitive ability.

Second, one SNP, rs9765600, was significantly negatively associated with numerical ability (Aurora-N) as well as verbal ability (Aurora-V). This SNP is located in an intron of proline-rich 16 also known as Largen (*PRR16*; located on chromosome 5q23.1), a cell size regulator that promotes cell size increase through the upregulation of messenger RNAs, in particular those involved in mitochondrial functions (Yamamoto and Mak, 2017).

Third, Aurora-N was also nominally associated with intronic rs2806225 located in the *DSP* (desmoplakin, 6p24.3) gene and rs1863004 located in the CCR4-NOT transcription complex subunit 4 (*CNOT4*; 7q33) gene. Desmoplakin is a critical component of desmosome structure and helps maintain the structural integrity of cell–cell junctions. Mutations in *DSP* are associated with abnormal cell–cell junctions and cause cardiomyopathies and keratodermas. In the brain, DSP is highly and specifically expressed in the dental gyrus, suggesting that it plays a role in hippocampal neurogenesis (Wang et al., 2015), possibly through interactions with cadherin family proteins. CCR4-NOT complex is a highly conserved transcriptional regulator that is essential for neural development (Chen et al., 2015) and differentiation of neural stem cells and has been featured in studies of the 7q33 deletion syndrome (Lopes et al., 2018) that is characterized by intellectual disability.

### Gene-Based Genome-Wide Association Study of Aurora-G Scores

**Figure 3:**
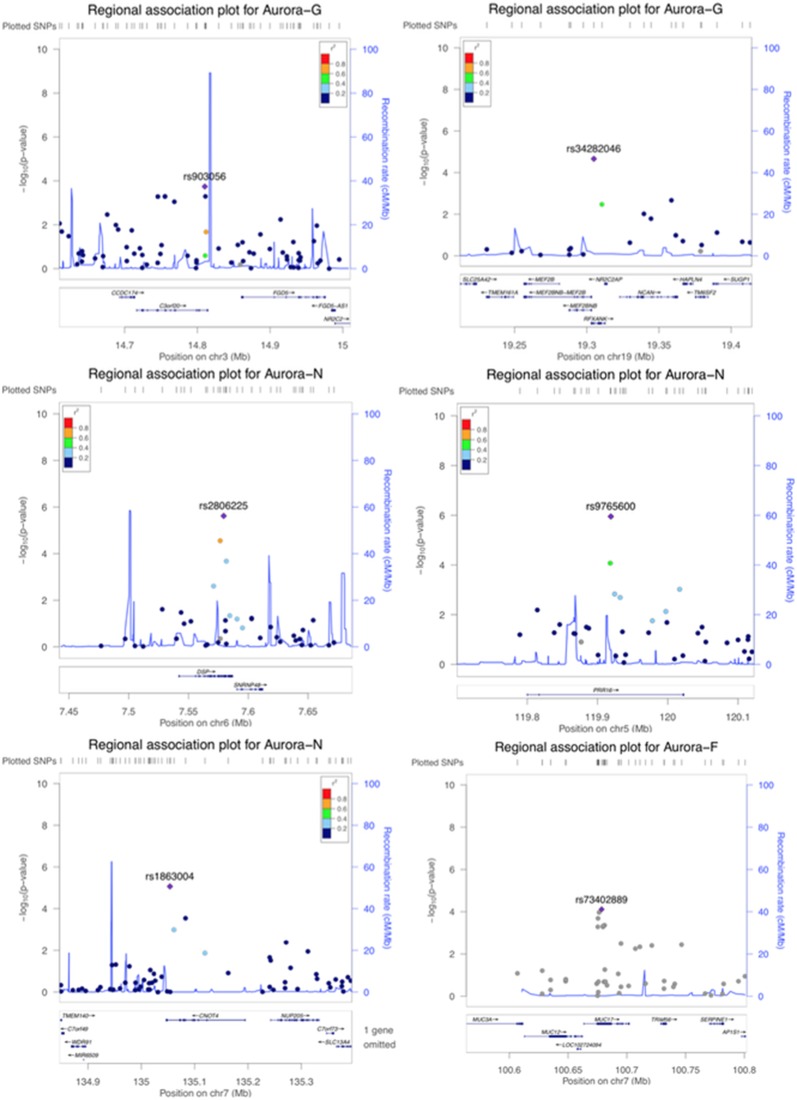
Regional association plots for genome-wide significant regions (gene-based analyses).

Although our SNP-based analyses did not reveal genome-wide significant associations that would survive corrections for multiple testing, gene-based association analyses performed in KGG revealed a more pronounced pattern of results. Six genes survived corrections for multiple testing (**Table 2**; regional association plots are presented in **Figure 3**). Three of these extend and replicate SNP-based findings: Aurora-N was associated with *PRR16* (*P* = 1.00 × 10^-6^), *CNOT4* (p = 1.39 × 10^-6^), and *DSP* (p = 1.74 × 10^-8^).

**Table 2:** Genome-wide significant gene-based associations for Aurora.

Gene	k SNPs	Gene-based *P*	Adjusted P	Chr	Size (bp)	Phen
*DSP*	18	1.74 × 10^-8^	0.00119789	6p24.3	45,143	Aurora-N
*C3orf20*	21	2.38 × 10^-7^	0.00819244	3p25.1	98,812	Aurora-G
*PRR16*	24	1.00 × 10^-6^	0.02294800	5q23.1	223,055	Aurora-N
*CNOT4*	8	1.39 × 10^-6^	0.02392329	7q33	148,333	Aurora-N
*MUC17*	22	1.98 × 10^-6^	0.02726222	7q22.1	38,788	Aurora-F
*NR2C2AP*	2	4.03 × 10^-6^	0.04624022	19p13.11	2,027	Aurora-G

K SNPs, number of SNPs assigned to the gene for the association analysis; Adjusted p, p-value adjusted using the Benjamini–Hochberg correction for multiple comparisons (applied for all phenotypes and genes). Phen, phenotype.

We also established a genome-wide significant association between general cognitive ability (Aurora-G) and two additional genes—*C3orf20* (chromosome 3 open reading frame 20 (3p25.1) at *P* = 2.38 × 10^-7^ and *NR2C2AP* (nuclear receptor 2C2 associated protein; 19p13.11) at *P* = 4.03 × 10^-6^. Although the function of the encoded protein is unknown, *C3orf20* maps onto the locus that has been associated with age of onset of sporadic Alzheimer’s disease (Velez et al., 2016). Notably, this locus, as well as *C3orf20* itself, was recently implicated in recessive intellectual disability (Kariminejad et al., 2015) in three siblings of Iranian ancestry.

The second significant signal for Aurora-G comes from the locus containing the gene encoding NR2C2-associated protein (*NR2C2AP*), which is hypothesized to repress NR2C2-mediated transactivation through the suppression of binding between NR2C2 and TR4-response elements in target genes. The signal for *NR2C2AP* is driven by two SNPs assigned to this gene—rs10424365 (*B* = -4.25, *SE* = 1.44, *P* = 0.003319076; MAF = 13.4%) and rs34282046 (*B* = -16.19, *SE* = 3.76, *P* = 2.17 × 10^-5^; MAF = 1.8%). Both SNPs were also assigned to regulatory factor X associated ankyrin containing protein (*RFXANK*), a transmembrane protein from the MHC class II family that is involved in MHCII expression, is regulated by class IIa histone deacetylases (Wang et al., 2005), and was highlighted as a transcriptional regulator of neural stem/progenitor cells in mice (Kawase et al., 2014); rs34282046 is a nonsynonymous mutation that affects 9 alternative *RFXANK* transcripts, yet is predicted to be benign by PolyPhen2 (Score = 0.000).

Finally, Aurora-F was associated with mucin 17 (*MUC17*, cell surface associated; 7q22.1) at *P* = 1.98 × 10^-6^. The protein encoded by this gene is mostly found in epithelial cells of the intestinal tract. *MUC17* is one of the over 100 genes recently reported to be included in the interstitial deletion of the 7q22.1 syndrome, which is characterized by structural brain abnormalities and intellectual disability (Katz et al., 2016). We did not establish any genome-wide significant associations for Aurora-V.

### Genome-Wide Surrogate Runs of Homozygosity Association Analysis

**Figure 4:**
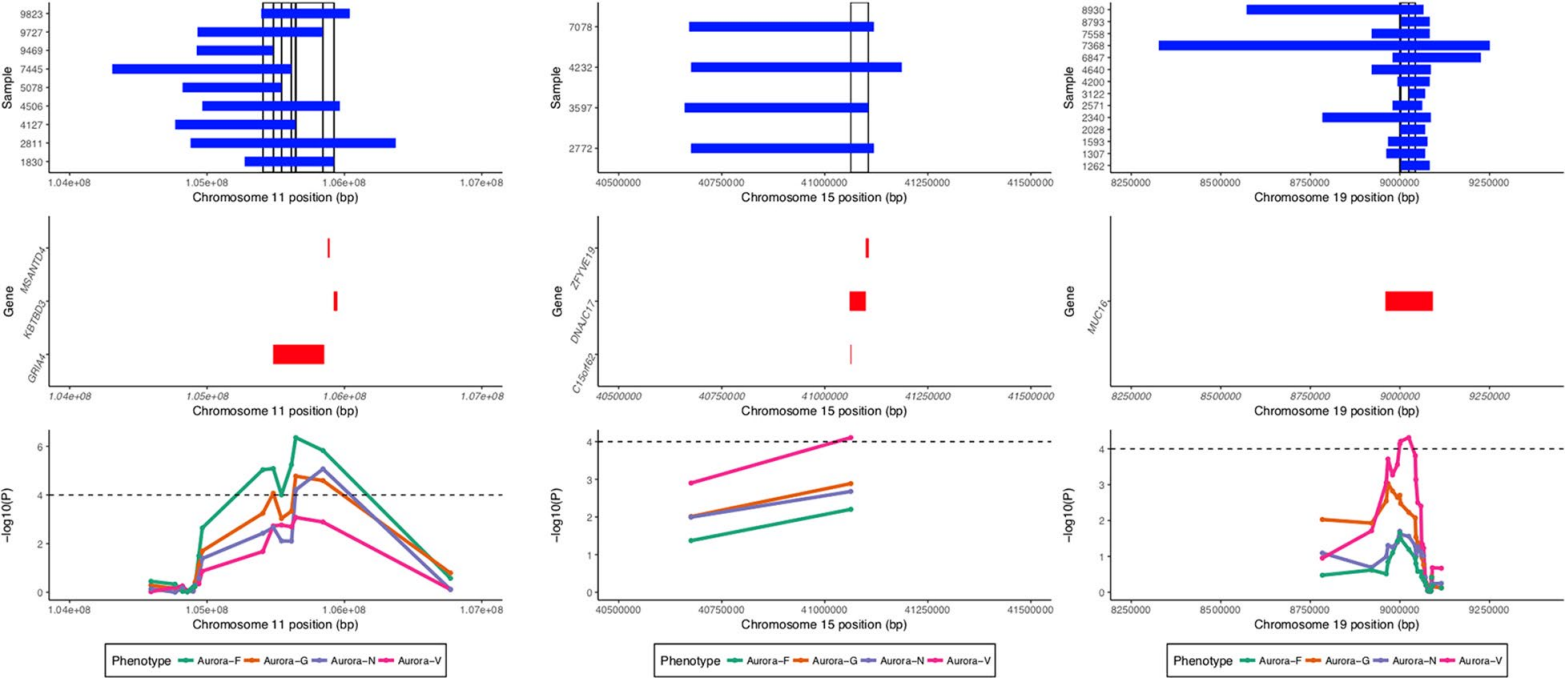
Regional association and sROH visualization plot. Top panel shows ROHs present in individual samples in regions significantly associated with the phenotypes. Vertical bars denote surrogate ROHs tested for association. Middle panel displays genic regions for the three plots. Bottom panel displays curves for the association signal for different phenotypes for the tested sROHs.

ROHs identified by GARLIC were subjected to two complementary analyses: homozygosity burden analyses using f_ROH_ estimates and ROH association analyses using a binary matrix of surrogate ROHs (sROHs) that accounted for the partial overlaps of ROHs in the sample. The analyses were performed using custom R scripts. The identification of surrogate ROHs followed the algorithm parallel to that employed by the CNVRuler (Kim et al., 2012) software for the detection of overlapping CNV segments; the two approaches produced an identical set of ROH regions. Given the methodological considerations of superficial signal similarity between ROHs and hemizygous deletions (Mcquillan et al., 2008), we conservatively restricted the set of ROHs in the association analyses to those that did not overlap with copy number variable regions detected by PennCNV for the entire study sample (see **Figure 4**).

K = 30,733 unique sROHs outside of CNV regions were called, and 19,214 had a frequency above 1% (at least 4 out of 354 individuals in the sample). sROH frequency was inversely correlated with its length at ρ = -0.15 (*p* < 0.001). To reduce the number of multiple comparisons and increase power, further analyses focused specifically on k = 11,992 genic sROHs that we hypothesized were more likely to have an interpretable pronounced phenotypic effect than intergenic sROHs.

Four loci exhibited a total of 15 sROH segments with nominal association P values < 0.0001 (see **Table 3**). A region on chromosome 11q22.3 survived corrections for multiple testing (for Aurora-F, P_adj_ = 0.02093395 and 0.03569284 for the two sROHs) and was simultaneously negatively associated with Aurora-G and positively—with Aurora-F and Aurora-N. Effect sizes exceeded 20 standard IQ points, and allele frequencies ranged from 1.12 to 2.54%, with the cumulative length of the affected region estimated at around 796 kb. This region contains several important genes, most notably glutamate ionotropic receptor alpha-amino-3-hydroxy-5-methyl-4-isoxazolepropionic acid type subunit 4 (*GRIA4*), an L-glutamate receptor gene that is highly expressed in the central nervous system and is involved in excitatory synaptic transmission. We further interrogated the sample for the presence of possible coding mutations in *GRIA4* by performing genotype imputation using BEAGLE (Browning and Browning, 2009) and a custom reference panel constructed using whole-genome DNA sequencing data from n = 108 individuals of Qatari ancestry (k = 9,763,073 markers) (Rodriguez-Flores et al., 2016). Using this dataset, we were able to impute five coding SNPs in the study sample (rs57375419, rs7933212, rs56091908, rs116000289, and rs61736488). All of the ROH carriers in the study sample were homozygous for the reference alleles of the imputed SNPs.

**Table 3:** Nominally significant (at P < .0001) associations between sROHs and Aurora.

sROH	*B*	*SE*	*T*	*P*	Adjusted *P*	Chr	Start	End	Length	Carriers	Gene	Phen
sROH_17583	22.20	4.93	4.50	9.19 × 10^-6^	0.073441885	11	105,405,895	105,482,024	76,130	9/354	*GRIA4*	Aurora-F
sROH_17584	-20.89	5.26	-3.98	8.53 × 10^-5^	0.292376267	11	105,482,025	105,541,743	59,719	8/354	*GRIA4*	Aurora-G
sROH_17584	23.64	5.22	4.53	8.20 × 10^-6^	0.073441885	11	105,482,025	105,541,743	59,719	8/354	*GRIA4*	Aurora-F
sROH_17585	22.10	5.61	3.94	9.89 × 10^-5^	0.316240857	11	105,541,744	105,614,894	73,151	7/354	*GRIA4*	Aurora-F
sROH_17586	27.68	6.01	4.61	5.69 × 10^-6^	0.073441885	11	105,614,895	105,645,648	30,754	6/354	*GRIA4*	Aurora-F
sROH_17587	-28.75	6.59	-4.36	1.69 × 10^-5^	0.115640555	11	105,645,649	105,844,065	198,417	5/354	*GRIA4*	Aurora-G
sROH_17587	26.82	6.61	4.05	6.18 × 10^-5^	0.269457038	11	105,645,649	105,844,065	198,417	5/354	*GRIA4*	Aurora-N
**sROH_17587**	**33.59**	**6.52**	**5.15**	**4.36** × **10^-7^**	**0.020933945**	**11**	**105,645,649**	**105,844,065**	**198,417**	**5/354**	*GRIA4*	Aurora-F
sROH_17588	33.18	7.34	4.52	8.52 × 10^-6^	0.073441885	11	105,844,066	105,923,564	79,499	4/354	*KBTBD3, GRIA4, MSANTD4*	Aurora-N
sROH_17588	-31.42	7.37	-4.27	2.57 × 10^-5^	0.153851824	11	105,844,066	105,923,564	79,499	4/354	*KBTBD3, GRIA4, MSANTD4*	Aurora-G
**sROH_17588**	**35.79**	**7.31**	**4.90**	**1.49** × **10^-6^**	**0.035692842**	**11**	**105,844,066**	**105,923,564**	**79,499**	**4/354**	*KBTBD3, GRIA4, MSANTD4*	Aurora-F
sROH_20937	29.54	7.39	4.00	7.77 × 10^-5^	0.286862153	15	41,063,245	41,105,658	42,414	4/354	*DNAJC17, C15orf62, ZFYVE19*	Aurora-V
sROH_23251	-18.06	4.50	-4.01	7.30 × 10^-5^	0.286862153	19	8,999,675	9,001,832	2,158	11/354	*MUC16*	Aurora-V
sROH_23252	-16.82	4.15	-4.05	6.18 × 10^-5^	0.269457038	19	9,001,833	9,024,870	23,038	13/354	*MUC16*	Aurora-V
sROH_23253	-16.46	4.00	-4.11	4.85 × 10^-5^	0.258308827	19	9,024,871	9,043,020	18,150	14/354	*MUC16*	Aurora-V

sROH, unique surrogate ROH identifier; B, effect size (in standardized scores units); SE, standard error; t, t-value for regression coefficient. Adjusted P—p-value adjusted using the Benjamini–Hochberg correction. Adjusted P-values < 0.05 are shown in **bold**. Phen, phenotype.

Finally, Aurora-V was positively associated with sROH located on chromosome 15q15.1 and encompassing DnaJ heatshock protein family (Hsp40) member C17 (*DNAJC17*), also involved in autoimmune disorder ileocolitis, as well as with chromosome 15 open reading frame 62 (*C15orf62*), and zinc finger FYVE-type containing 19 (*ZFYVE19*).

### Contribution of Genome-Wide Homozygosity and CNV Burden to Cognitive Ability

In order to capture inter-individual variation in patterns of homozygosity and structural variant burdens, we conducted a LCA using f_ROH(A)_, f_ROH(B)_, f_ROH(C)_, f_SNP_, and CNV burden values as indicator variables. Analysis of BIC values for the estimated models for k latent mixture components ranging from 1 to 10 suggested that the best fit is provided by the five-component model that postulates the presence of five latent classes of individuals (additional sensitivity analyses using reduced variable groupings and alternative formulations of models converged on a five-mixture model; data not shown). The respective model displayed EVE geometric notation (ellipsoidal distribution, equal volume, and variable shape among latent classes); for details, see Browne and Nicholas (Browne and Mcnicholas, 2014) and **Supplementary Material**. Model fit was also evaluated using sequential bootstrap likelihood ratio test with 1,000 bootstrap samples: the analyses suggested that model fit improved with increases in number of estimated mixture components from 1 to 2 (p = 0.000999001), from 2 to 3 (p = 0.00099001), from 3 to 4 (p = 0.001998002), and from 4 to 5 (p = 0.000999001) but not from 5 to 6 (p = 0.946053946), suggesting that the k = 5 mixture components model indeed provides best fit to the data.

The classes ranged in size from 11 to 181 individuals and could heuristically (based on the results of permutation-based analysis of variance for indicator variables; data not shown) be labeled according to **Table 4**, which provides relative qualifiers for estimated SNP and ROH homozygosity and CNV burden. Overall, 51% of the sample was classified into Class 3, characterized by low levels of homozygosity and CNV burden. Classes 1 and 2 had moderate levels of homozygosity but were characterized by increased CNV burden. Classes 4 and 5, on the other hand, represented individuals with low CNV burden but dramatically elevated f_ROH_ homozygosity driven by class B and C ROHs, respectively.

**Table 4:** Heuristic description of five latent classes estimated using ROH and CNV burden values.

Latent Class	N	f_SNP_			f_ROH_			CNV burden		
			*M*	*SD*		*M*	*SD*		*M*	*SD*
1	11	Moderate	0.040	0.030	Moderate	3.26%	1.22%	High	0.218%	0.182%
2	25	Moderate	0.032	0.039	Moderate	4.97%	2.56%	Moderate	0.096%	0.035%
3	181	Low	0.008	0.032	Low	1.79%	0.79%	Low	0.018%	0.016%
4	69	High	0.064	0.056	High	7.48%	4.18%	Low	0.008%	0.008%
5	68	High	0.086	0.032	High	6.98%	2.55%	Low	0.022%	0.016%

**Figure 5:**
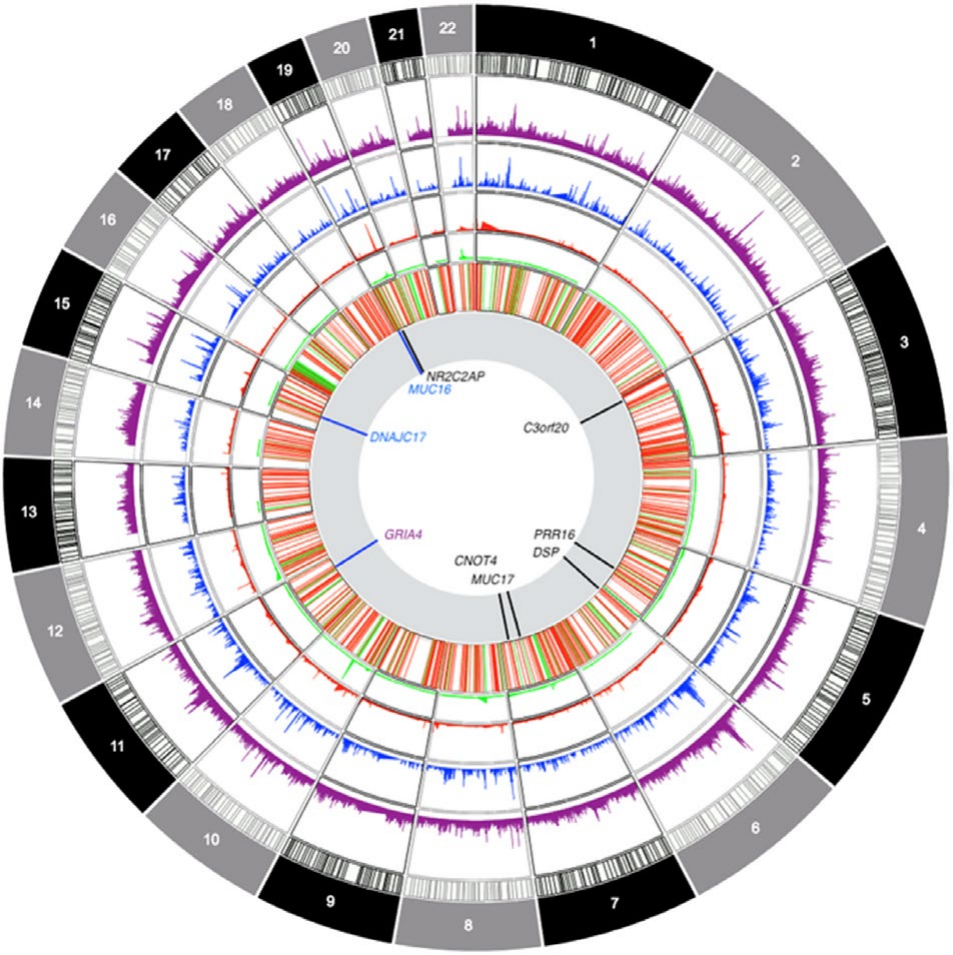
Contribution of fROH and CNV burden variables to general cognitive ability (Aurora-G) in four latent classes of individuals. Top panel presents data for Class1 + Class2, bottom panel—for Class4 + Class5.

Notably, although the five latent classes did not differ with respect to average estimated cognitive ability (all *P*’s > 0.6667; see Supplementary Material), they differed in patterns of association between cognitive ability and fROH and CNV burdens (**Figure 5**). For the purpose of the analysis, we combined Classes 1 and 2 into one group. Since the primary focus of our analyses was on general cognitive ability, Aurora-G was used as the main phenotype for the association analyses. All analyses controlled for the first five principal components estimated from genotyping data.

Given the small sample size for Classes 1 + 2, we used simple linear regression with permutation testing to evaluate the contribution of fROH and CNV burden to cognitive ability in this group. Analyses indicated that although the model with just PC covariates was not statistically significant, *F*_(5,30)_ = 1.791, *P* = 0.145, *R^2^* = 0.1015, adding fROH and CNV burden variables drastically improved its fit, *F*_(9,26)_ = 3.395, *P* = 0.006991, *R^2^* = 0.3812, with fROH and CNV burden explaining an additional 28% of variance in general cognitive ability in this sample, with statistically significant negative contributions from f_ROH(c)_, *B* = -2.21, *P* = 0.044, and CNV burden, *B* = -49.57, *P* =.001. Thus, in this subgroup of individuals with moderate ROH and substantial CNV burdens, both variables accounted for over a quarter of the variation in cognitive ability. However, sensitivity analyses removing one outlying data point rendered the CNV burden effect nonsignificant while preserving the f_ROH(c)_ effect.

Notably, in other groups, we did not establish significant contributions of ROH and CNV burdens to cognitive ability, using linear regression fit for the entire distribution of ability values (data not shown). Given the larger sample size of the combined Classes 4 and 5 and Class 3, we further investigated the relationship between ROH and CNV burdens and cognitive ability at different segments of its distribution, thereby accounting for possible nonlinear effects. Thus, quantile regressions were fit separately for Class 3 (n = 181) and Classes 4 and 5 (n = 137). Parameter estimates can be found in the **Supplementary Material**.

This analysis revealed that for Class 3, the only significant effect was observed for f_ROH(B)_ at the 50^th^ quantile (*B* = 6.22, *t* = -2.51, *P* = 0.01314), with higher homozygosity due to medium-length ROHs being associated with lower cognitive ability. At the same time, for Classes 4 and 5, it was the f_ROH(C)_ burden that acted as a significant negative predictor of Aurora-G at the 25% quantile (*B* = -1.28, *SE* = 0.60, *t* = -2.11, *P* = .03664) but not 50% quantile (*B* = -.14, SE = 0.42, *t* = -0.32, *P* = .15322) and 75% quantiles (*B* = -.14, *SE* = 0.42, *t* = -0.32, *P* = .15322). In Classes 4 and 5, CNV burden was also a strong negative predictor of cognitive ability at the 75-th quantile (*B* = -214.64, *SE* = 74.96, *t* = -2.86, *P* = 0.00491).

Thus, our regression analyses performed separately for three groups of individuals suggested that in the presence of substantial f_ROH_ and CNV burdens, these variables are strongly predictive of general cognitive ability, with decreases in Aurora-G scores estimated at 2.21 standardized IQ points for each 1% increase in f_ROH(C)_ and almost 50 points for each 1% increase in CNV burden. In the class characterized by low levels of CNV and ROH burdens, homozygosity burden due to medium-length ROHs predicted ability negatively in the average ability range. In the classes of individuals characterized by high levels of CNV and ROH burdens, homozygosity effects were due to consanguinity-related long ROHs, and effects were pronounced in the lower and higher but not the median portion of the cognitive ability distribution.

## Discussion

In this study, we examined the genetic underpinnings of cognitive ability in a sample of children from the consanguineous population of Saudi Arabia. First and foremost, we demonstrated that the homozygosity burden in this population is significant, with approximately half of the sample estimated to have f_ROH_ burden values above 3%. Even individuals in Class 3 characterized by relatively low levels of f_ROH_ still had an estimated f_ROH_ at 1.79%; this is over 10 times larger than the lowest average level of f_ROH_ across European populations presented in a recent study (Howrigan et al., 2016) that evaluated contributions of genome-wide homozygosity to cognitive ability. At the same time, 49% of the individuals in our sample were identified as demonstrating moderate to high levels of inbreeding, with the average f_ROH_ estimated in Class 4 at 7.48% of the autosomal genome, i.e., over 12 times larger than the highest f_ROH_ value for populations studied previously.

We established that individuals with low and high levels of CNV and ROH burden did not differ with respect to their average cognitive ability. The absence of group differences in cognitive ability between children with low and high homozygosity burden estimates in the sample is intriguing. It is possible that the potential performance gap between these classes of children is masked by the advantageous effects of the high socioeconomic status of children born into the arranged, consanguineous marriages of the Saudi traditional families, which are well supported both financially and via social services (Warsy et al., 2014). Nonetheless, when limited to the analysis within this subpopulation, fROH and CNV burdens negatively predicted cognitive ability at its lowest and highest distributional tails in the subsample with pronounced CNV and ROH burden, with the longest C-class ROHs driving the effect. This class of ROHs is hypothesized to arise through parental relatedness and has been noted to contain a disproportionate number of detrimental homozygous variants (Szpiech et al., 2013), directly linking consanguinity in the studied population with variation in children’s cognitive ability.

Homozygosity mapping in the current study also identified a subgroup of nine individuals carrying a ROH that encompasses the *GRIA4* gene. Out of nine carriers of ROHs in this region, the vast majority (8/9) were assigned to groups with pronounced ROH burden, and the presence of ROHs in this region was associated with lower general cognitive ability and higher levels of the figural ability, effectively indexing disparate cognitive profile with lower estimated general ability (ranging from 78 to 122 for carriers, *M* = 93.89; *SD* = 18.93) but higher spatial ability (ranging from 88 to 145 for carriers, *M* = 121.80; *SD* = 19.41). Given the higher reliability of the general cognitive ability estimate due to its reliance on nine indicator variables (as opposed to three residual indicators for domain factors), we believe this effect should be interpreted as capturing variance related primarily to general cognitive ability.

*GRIA4* encodes a subunit of the alpha-amino-3-hydroxy-5-methyl-4-isoxazolepropionic acid receptors that underlie excitatory synaptic transmission and activity-dependent synaptic plasticity (Bettler and Fakler, 2017) and has been implicated in a range of human phenotypes, including schizophrenia (Marshall et al., 2017), substance use, and major depression. In a recent exome sequencing study of individuals with intellectual disability, *de novo* heterozygous pathogenic variants in *GRIA4* were found in five unrelated individuals with the condition (Martin et al., 2017). Although the functional impact of the identified ROHs on *GRIA4* function in the Saudi population is unknown, these genome-wide significant findings nonetheless suggest that this gene plays a role in the regulation of cognitive traits in the population. Notably, *GRIA4* was highlighted as a significant predictor of cognitive ability in the largest meta-GWAS of cognitive ability to date (Davies et al., 2018).

Of note is that, homozygosity mapping also identified a locus on chromosome 19 harboring the *MUC16* gene as being nominally associated with verbal ability in this sample. Although this locus did not survive corrections for multiple testing, this finding is noteworthy for two reasons. First, it corresponded to the ROH hotspot on chromosome 19 clearly visible in **Figure 4**: 14 out of 354 (or 4%) individuals had a ROH in this region. Although increased regional homozygosity has been previously associated with positive selection, in our sample, the average verbal general cognitive ability among carriers of ROH was estimated at 86.97 IQ points, slightly above the -1 SD cutoff, with 8 out of 14 individuals displaying scores at or below 85 standard points. Second, another related gene, *MUC17*, was genome-wide significantly associated with figural/spatial ability in this sample. The lead SNP from this gene was rs73402889, a missense S>I mutation in exon 3 of *MUC17* that is predicted to be tolerated by SIFT. This SNP also is a significant expression QTL for *CLDN15* that encodes Claudin-15, which is an integral membrane protein that is involved in the structure and maintenance of tight junctions and cell polarity. Both MUC16 and MUC17 proteins belong to the class of transmembrane mucins containing SEA domains. Transmembrane mucins are highly expressed in epithelial cells. In the developing brain, MUC16 and MUC17 are both expressed at 10 weeks post-conception in the choroid plexus (Expression Atlas; https://www.ebi.ac.uk/gxa/), a network of blood vessels and cells involved in the production of cerebrospinal fluid. It is possible that transmembrane mucins contribute to cognitive ability in this population through their involvement in mucin-type O-glycosylation, a form of posttranslational protein modification that promotes vascular integrity (Herzog et al., 2014) and are involved in a range of developmental processes. Studies suggest that O-glycosylation is involved in the stabilization of early brain vasculature and that subventricular zones containing neural progenitor cells are particularly susceptible to O-glycosylation deficiencies (Xia et al., 2004).

**Figure 6:**
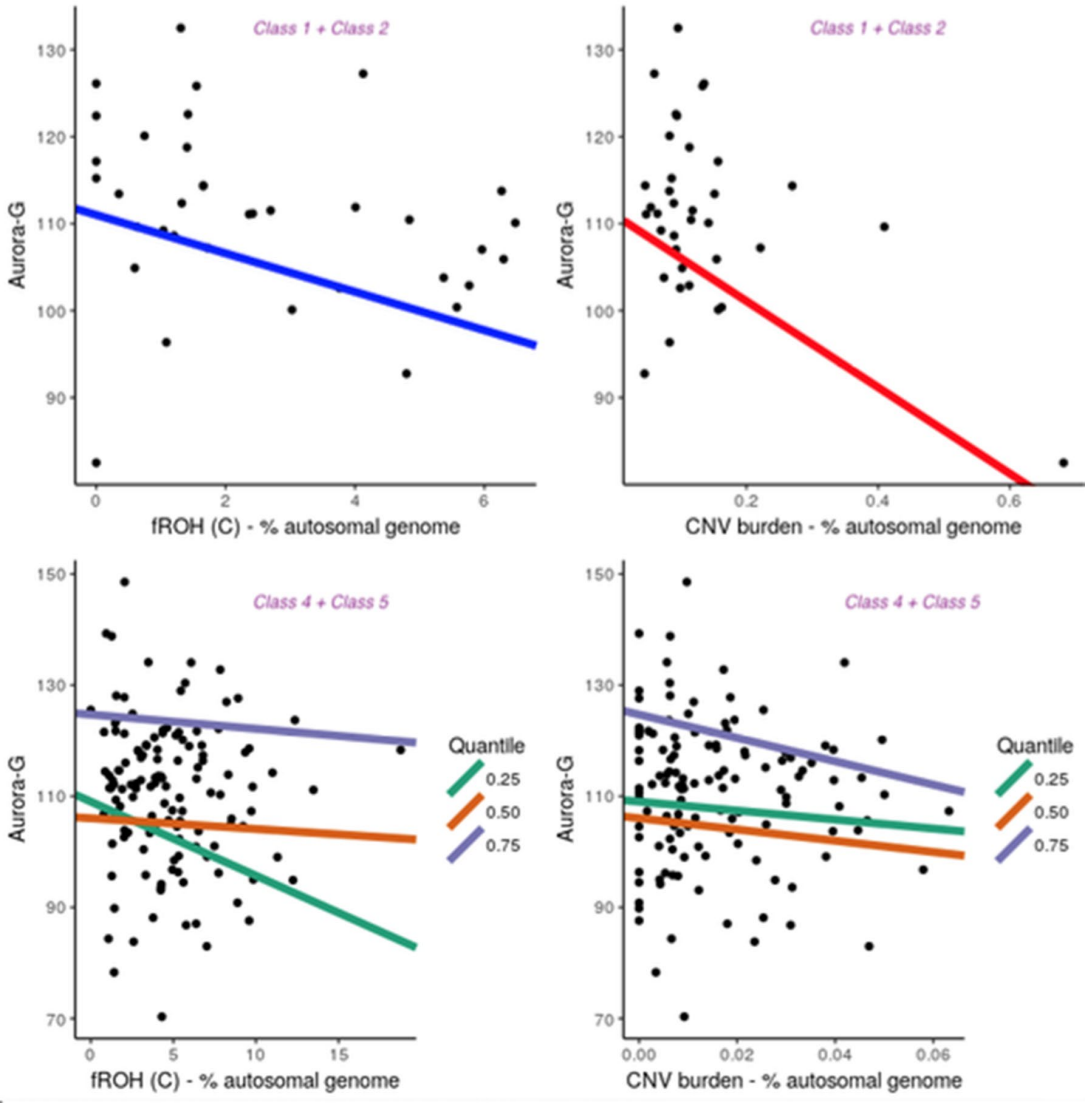
Circos-style plot of genome-wide association study data integrating gene-based and ROH-based findings. Starting from the outside, tracks denote: frequency of sROH segments prior to CNV filtering, frequency of sROHs outside CNV regions, frequency of CNV loss events, frequency of CNV gain events, joint distribution of CNV segments (regions excluded from ROH analyses), association results. The last track highlights genomic locations with findings that survived multiple corrections for gene-based association tests (in black) and findings at *p* < 0.0001 for sROH association analyses (blue) as well as the finding from the *GRIA4* (purple) region that survived corrections for multiple testing.

The genes highlighted in this study (see **Figure 6** for a summary) did not harbor pathogenic mutations in the clinical exome study of a primarily pediatric cohort of 454 phenotypically heterogeneous probands (Alfares et al., 2017) or an even larger study of over 1,000 families also with heterogeneous and suspected Mendelian phenotypes (Monies et al., 2017) from Saudi Arabia, as well as a smaller cohort of 149 children and young adult probands from Qatar (Yavarna et al., 2015). This is not contrary to our findings, as we selected our sample for higher cognitive ability scores in the population sample (i.e., the opposite of the selection criteria for the abovementioned studies). Yet, at this point, it is important to attempt a replication of these findings in an unselected sample to verify the generalizability of the obtained results. Interestingly, our findings do overlap with genes identified in recent meta-analyses of cognitive ability: in addition to the *GRIA4* finding, we established genome-wide significant associations for *CNOT4* and *PRR16*, both of which are highly expressed in the brain and have been previously associated with cognitive ability (Davies et al., 2018).

Saudi Arabian researchers are actively examining the effects of consanguinity on the prevalence and etiology of Mendelian traits, and recent studies have been extremely fruitful in identifying coding variants responsible for fairly severe phenotypes, including intellectual disability. The results of our study are in concert with the growing realization of the importance of ROH in a variety of traits and conditions (Lencz et al., 2007; Yang et al., 2010; Keller et al., 2012; Gamsiz et al., 2013; Power et al., 2014a; Power et al., 2014b; Verweij et al., 2014; Gandin et al., 2015; Ghani et al., 2015; Mezzavilla et al., 2015; Howrigan et al., 2016) and highlight ROHs as population-specific alleles that form the genetic foundation for individual differences in cognitive ability. This complex trait regulation from genetic variants that can harbor multiple deleterious variants in the homozygous forms suggests that ROHs represent an important class of genetic variants and that theoretical developments in human trait genetics should attempt to incorporate consanguinity and ROHs [for example, from the infinitesimal model (Barton et al., 2017)] into their models. It has been noted that age-related Flynn effects (i.e., substantial and sustained population-level increases in cognitive ability over time) are smaller in Arab countries (Bakhiet et al., 2018); it is possible that in addition to cultural environmental factors (such as educational standards) highlighted by others, such disproportionally small cognitive ability gains over time can be attributed to population-specific genomic background characterized by elevated homozygosity as a substantial risk factor.

Admittedly, the key limitation of the present study lies in its small sample size (in fact, it is 1,000 times smaller than the most recent publication on the genetics of cognitive ability) and the absence of a replication sample. We would like to note, however, that this study yielded a pronounced pattern of interpretable results, both in terms of quantifying the extent of homozygosity burden in children in Saudi Arabia and highlighting its substantial contribution to the genetic foundation of cognitive ability in this consanguineous population. Pathway enrichment analyses (**Supplementary Material**) indicated two overrepresented pathways (cadherin and Wnt-signaling) and also highlighted calcium ion binding and nervous system development as over-represented molecular function and biological process terms. Homozygosity mapping implicated *GRIA4* and *MUC16* as possible regulators of cognitive ability in this sample, with the majority of individuals harboring the ROHs encompassing these genes being assigned to the high homozygosity burden latent classes, thereby linking consanguinity, homozygosity, and cognitive ability. To the best of our knowledge, this is the first investigation of the genetic foundation of cognitive ability of a highly consanguineous population of Saudi Arabia, and replication datasets are lacking at the moment.

Future studies could utilize recently published clinical microarray and sequencing data produced by the multicenter clinical team in KSA, which contains a sizeable number of individuals with neurodevelopmental disorders (Alfares et al., 2017; Monies et al., 2017). Given the demonstrated power of the approach taken by this study, future studies should also attempt further characterizing the contribution of homozygosity burden to cognitive ability in highlighted consanguineous populations, such as populations from Central and South Asia (Pemberton and Rosenberg, 2014), as homozygosity and CNV burdens were associated with variation in cognitive ability, specifically in groups where the genetic load of these factors was substantial.

## Data Availability

The data generated for this study can be found in the dbGAP NCBI repository using accession number phs001884.v1.p1 (http://www.ncbi.nlm.nih.gov/projects/gap/cgi-bin/study.cgi?study_id=phs001884.v1.p1).

## Ethics Statement

The study protocol and procedures were approved by the Internal Review Boards of Yale University and University of Houston. Parents of children participating in the study and authorities at the appropriate educational institutes provided written informed consent in accordance with the Declaration of Helsinki.

## Author Contributions

EG, MT, and AA conceptualized and designed the study and the study assessments and supervised the data collection. ON and SK processed the biological specimens. SK, ON, and EG planned and carried out the analyses. SK, MT, AA, ON, and EG drafted, revised, and approved the final manuscript.

## Funding

This research was supported by the Ministry for Higher Education, Kingdom of Saudi Arabia. Grantees undertaking such projects are encouraged to express freely their professional judgment. The paper, therefore, does not necessarily reflect the position or policies of the previously mentioned funding agencies, and no official endorsement should be inferred.

## Conflict of Interest Statement

The authors declare that the research was conducted in the absence of any commercial or financial relationships that could be construed as a potential conflict of interest.
