# Supplementary material for: Genome-Wide Homozygosity Mapping Reveals Genes Associated With Cognitive Ability in Children From Saudi Arabia
Source: Front Genet. 2019 Sep 18;10:888. doi: 10.3389/fgene.2019.00888 (PMC6759945; doi:10.3389/fgene.2019.00888)
Supplement: Supplementary file 1 [file DataSheet_1.pdf]

Supplementary Material

Genome-wide Homozygosity Mapping Reveals Genes Associated with Cognitive Ability in Children from Saudi Arabia

Sergey A. Kornilov, Mei Tan, Abdullah Aljughaiuman, Oksana Yu. Naumova, Elena L. Grigorenko*

*** Correspondence:** Corresponding Author: elena.grigorenko@times.uh.edu

1 Supplementary Analysis of Background Relatedness in the Study Sample

Additional analyses were undertaken to ensure that the results are stable to the presence of closely related individuals in the sample – i.e., although mixed linear modeling adequately represented sample structure, including both admixture and relatedness, analyses of sROH associations were carried out using principal component regression, and analyses of ROH and CNV burdens' contribution to cognitive ability were modeled using quantile regression. Correspondingly, we evaluated sample relatedness using a combination of techniques.

Post-QC sample genotypes were phased using BEAGLE v. 3 (Browning and Browning, 2009) with default settings. Genetic locations for SNP markers in cM were acquired from the Rutgers Combined Linkage-Physical Map v.3 (http://compgen.rutgers.edu/rutgers_maps.shtml) (Matise et al., 2007).

Relatedness was evaluated using two complimentary approaches. Phased genomes were subjected to Identity by Descent (IBD) estimation using FISHR2 (Bjelland et al., 2017), a recently developed algorithm for detection of IBD segments between individuals from genome-wide SNP data that utilizes a modified version of GERMLINE (Gusev et al., 2009) as an initial screen for candidate IBD segments and then stitches together contiguous segments separated artificially due to SNP or phase errors while differentiating between locations possibly inconsistent with IBD inheritance due noise in the data as opposed to lack of truly IBD signal.

Analyses began with the identification of long (> 2.0 cM) segments with a minimum of 25 SNPs detected with a window of 50 and a gap parameter of 5 markers and FISHR2 manual-recommended threshold parameters for error values. FISHR2 identified 158,973 IBD segments in the data, ranging from 2 to 40.96 cM in length, with the vast majority encompassing less than 5 cM (Mean = 2.56, SD = 0.85).

We then estimated pairwise relatedness using IBD2 segment data obtained via FISHR2 using ERSA 2.1 (Huff et al., 2011). ERSA is a maximum-likelihood method for estimation of recent shared ancestry and can accurately infer relatedness between pairs of individuals with power of nearly 100% for close relatives (1st through 5th-degree) and substantial power to detect even more distant relationships. Analyses were performed using default ERSA settings.

ERSA identified 41 unique pairs of relationships between 34 individuals total: 11 third-degree, 1 fourth-degree, 7 fifth-degree, 10 sixth-degree, and 12 seventh-degree relatives. Given the limited

resolution of the array and lack of genetic material sharing between very distant relatives, individuals identified as having 8-th and 9-th degree relatives were considered unrelated. Figure S6 and S7 shows the distributions of IBD segment sharing in unrelated vs related individuals in the sample.

Sensitivity analyses revealed that:

1) relatedness did not affect latent class analysis results, and no class was overrepresented among related individuals in the sample (among related individuals, n=1 belong to Class 1, n=4 to Class 2, n=11 to Class 3, n=6 to Class 4, and n=8 to Class 5);

2) there were no related individuals among carriers of sROHs reported in the main section of the manuscript as being associated with cognitive ability;

3) removing related individuals reduced the statistical significance of the $f_{ROH(C)}$ effect for Classes 1 and 2 to a marginal value of p=0.0715;

4) removing related individuals did not affect the pattern of the quantile regression solutions for Class 3 as well as for the combined Class 4 and 5.

2 Supplementary Analysis of Copy Number Variation Association with Cognitive Ability in the Study Sample

Association analysis was performed using the approach parallel to that of ROH analyses reported in the main text of the article – i.e., sepately for gain and loss events, we computed a set of disjoint surrogate CNV fragments (sLOSS and sGAIN for loss and gain events, respectively) that accounted for partial overlaps between called segments in the sample.

We specifically focused on CNVs that overlapped with known protein-coding genes (242 sGAIN segments, 842 sLOSS segments). Association with covariate and PC-adjusted phenotypes was performed using linear regression, and P-values were corrected for number of comparisons using the Benjamini-Hochberg's correction.

No surrogate CNV regions survived corrections for multiple testing (Manhattan plots and QQ plots are presented in Figures X and Y). After further reducing the number of multiple comparisons by excluding rare sCNVs present in less than 1% of the samples, no sCNVs (out of 100 sLOSS and 50 sGAIN segments) survived corrections for multiple testing.

Table S5 presents nominally significant sLOSS and sGAIN associations at uncorrected $p < 0.05$.

3 Supplementary Analysis of Ancestry in Children from Saudi Arabia

The analyses reported in the main text either adjusted phenotypes for five top genetic components estimated from the genetic data, or directly accounted for admixture by modeling pairwise kinship. Nonetheless, we performed an additional set of analyses aimed at providing more comprehensive information about the ancestral composition of the sample.

This analyses relied on the availability of two additional datasets: (1) a subset of n=176 individuals of Middle-Eastern ancestry (Bedouin, Druze, Mozabite, and Palestinian) from the Human Genome Diversity Project(Cann et al., 2002) (HGDP; data publicly available at http://www.hagsc.org/hgdp/files.html) that provides data on n=1,043 individuals of varying ancestry genotyped using Illumina's 650Y microarray panel; (2) a subset of the Qatar genome project dataset (Rodriguez-Flores et al., 2016) (DGMQ) that is comprised of whole genome-sequencing (WGS) data on n=108 unrelated natives of Arabian Peninsula, including n=56 of indigenous Arab ancestry (the data were graciously provided by Dr. Rodriguez-Flores from Cornell University).

For the HGDP dataset, we performed an additional QC of SNP markers following standard QC procedures using GoldenHelix SNP & Variation Suite. For the DGMQ dataset, VCF genotype calls were imported into SNP & Variation Suite and annotated against known dbSNP polymorphisms prior to further analyses. Ancestry evaluation was based on the set of k=147,057 SNP markers common to the two genotyping platforms that were also called in the DGMQ dataset and passed the QC in all three samples. Prior to the analyses, genotypes from the three different platforms were harmonized to the common marker map (we used the HumanCoreExome as the baseline map) to avoid strand alignment and genotype conversion ambiguity using Genotype Harmonizer (Deelen et al., 2014). Missing genotypes were imputed using the weighted k nearest neighbor approach (Schwender and Ickstadt, 2008) as implemented in the *scrime* package for R.

Ancestry evaluation was performed using two complimentary approaches. First, we applied the community-oriented network estimation (CONE) (Kuismin et al., 2017) method to the data. CONE is a recently developed method for ancestry and admixture evaluation that utilizes generalized linear model with LASSO regularization to infer relationships between individuals and populations from SNP-level data, and is a network-theory based method of population structure inference that does not rely on model parameters such as the prior number of subpopulations. Second, we used a maximum-likelihood (i.e., model-based) method for admixture analysis as implemented in ADMIXTURE (Alexander et al., 2009) software.

The neighborhood selection in CONE depends on the tuning/penalty parameter λ. Following Kuismin et al. (Kuismin et al., 2017) and Liu et al.(Liu et al., 2010), we chose to rely on the stability approach to regularization selection (StARS) to choose the most stable graph solution. StARS uses subsampling to measure uncertainty regarding the presence of edges between the nodes for each fixed value of λ. CONE performance was evaluated based on the examination of 100 different tuning parameter values in the range from 0.01 to 0.50 based on M=50 subsamples. λ value of 0.06210204 was chosen after the examination of the tuning parameter performance (Supplementary Figure S3), and Fruchterman-Reingold algorithm was used to detect different communities among the nodes. The results of CONE analyses (Supplementary Figure S4) suggested the presence of substantial genetic flow between the studied population of children from Saudi Arabia, and the majority of examined populations of Middle Eastern ancestry with the exception of Mozabite

(HGDP). The majority of the samples clustered with the Bedoin and Qatari samples, and substantial flow was also established for groups of samples that were related to the Druze and Palestinian populations.

We also analyzed the data using ADMIXTURE with 5-fold cross-validation for the range of k admixture components (ancestral populations) ranging from 2 to 10 using the block relaxation algorithm with Quasi-Newton convergence acceleration. Cross-validation (CV) values were used to guide model selection, and additional analyses were performed using StructureSelector(Li and Liu, 2018). CV values were estimated at 0.5949 for k=2, 0.59301 for k=3, 0.59266 for k=4, **0.59260 for k=5 (lowest)**, 0.59262 for k=6, 0.59307 for k=7, 0.59619 for k=8, 0.59714 for k=9, and 0.59758 for k=10. Population structure plots were obtained using StructureSelector's implementation of CLUMPAK(Kopelman et al., 2015) (see Supplementary Figure S5). The results of this analysis are consistent with the CONE-based results and suggest significant admixture in the presence of 5 ancestral populations, with the highest similarity observed between the study sample from Saudi Arabia and the sample of Qatar Genome Project (DGMQ)

4 Supplementary Enrichment Analysis of GO Terms and Pathways for Genes Associated with Cognitive Ability in the Study Sample

Enrichment analysis was performed for 2,542 genes identified in gene-based association analyses and 1,266 genes located within the sROHs that showed nominally significant ($p < 0.05$) evidence for association with any of Aurora ability scores using PantherDB (http://www.pantherdb.org). Statistical overrepresentation tests using Fisher's exact p-value estimation with FDR correction were computed for Panther pathways, GO molecular function and GO biological process terms.

1. **sROH-based associations ($p < 0.05$)**
   - **1.1. Pathway analysis** identified two over-represented pathways in sROH associations:
     - **1.1.1.** *cadherin signaling* pathway (3.60 fold enrichment, $P = 1.57 \times 10^{-9}$, $P_{FDR} = 2.56 \times 10^{-7}$) and
     - **1.1.2.** *Wnt-signaling* pathway (1.98 fold enrichment, $P = 1.89 \times 10^{-4}$, $P_{FDR} = 1.54 \times 10^{-2}$)
   - **1.2. GO molecular function** terms over-represented in the sROH gene list set
     - **1.2.1.** *calcium ion binding* (1.84 fold enrichment, $P = 8.68 \times 10^{-7}$, $P_{FDR} = 4.05 \times 10^{-3}$) and
     - **1.2.2.** *protein binding* (1.12 fold enrichment, $P = 3.82 \times 10^{-6}$, $P_{FDR} = 5.94 \times 10^{-3}$).
   - **1.3. GO biological process** analyses also revealed a significant over-representation of genes involved in
     - **1.3.1.** *hemophilic cell adhesion via plasma membrane adhesion molecules* (3.52 fold enrichment, $P = 4.40 \times 10^{-9}$, $P_{FDR} = 6.86 \times 10^{-5}$), and
     - **1.3.2.** *nervous system development* (1.40 fold enrichment, $P = 3.93 \times 10^{-6}$, $P_{FDR} = 2.04 \times 10^{-2}$)

2. **Gene-based associations ($p < 0.05$).**
   - **2.1. Pathway analysis** identified two over-represented pathways in gene-based associations:
     - **2.1.1.** *N/A*
   - **2.2. GO molecular function** terms under- and over-represented in the gene-based gene list set
     - **2.2.1.** *antigen binding* (0.18 fold enrichment, $P = 1.03 \times 10^{-6}$, $P_{FDR} = 9.58 \times 10^{-4}$) and
     - **2.2.2.** *protein binding* (1.11 fold enrichment, $P = 7.07 \times 10^{-10}$, $P_{FDR} = 8.24 \times 10^{-7}$).
   - **2.3. GO biological process** analyses also revealed a significant over-representation of genes involved in
     - **2.3.1.** *anatomical structure development* (1.15 fold enrichment, $P = 2.63 \times 10^{-5}$, $P_{FDR} = 3.73 \times 10^{-2}$)
       - **2.3.1.1.1.** *developmental process* (1.15 fold enrichment, $P = 3.18 \times 10^{-5}$, $P_{FDR} = 3.81 \times 10^{-2}$)
     - **2.3.2.** *multicellular organismal process* (1.13 fold enrichment, $P = 2.57 \times 10^{-5}$, $P_{FDR} = 4.00 \times 10^{-2}$)

2.3.3. *regulation of cellular process* (1.15 fold enrichment, $P = 4.62 \times 10^{-6}$, $P_{FDR} = 1.02 \times 10^{-2}$)
2.3.4. *cellular process* (1.09 fold enrichment, $P = 2.11 \times 10^{-12}$, $P_{FDR} = 1.10 \times 10^{-8}$)
2.3.5. *response to stimulus*(1.15 fold enrichment, $P = 5.22 \times 10^{-6}$, $P_{FDR} = 1.16 \times 10^{-2}$)
2.3.6. *adaptive immune response* (0.49 fold enrichment, $P = 2.90 \times 10^{-5}$, $P_{FDR} = 3.77 \times 10^{-2}$)

5 Supplementary Tables

Supplementary Table S1. Summary of study sample demographics and sampled geographic locations

	**Age**								**Region (n)**			
	Min	*Max*	*Med*	*Mean*	*SD*	n	Abha	Khamis-Mushyat	Tabuk	Al-Jubail	Jeddah	Al-Hassa
Boys	8.23	13.93	11.63	11.63	1.31	273	40	22	34	52	69	56
Girls	8.31	13.70	10.70	10.88	1.43	81	0	0	23	0	35	23
Total	8.23	13.93	11.42	11.33	1.36	354	40	22	57	52	104	70

Supplementary Table S2. Psychometric properties of Aurora-g subtests

Subtest	r_{xx}	% variance	K items	-LL
VA	0.66	15.3	17	-78253
VC	0.69	13.2	18	-78888
VS	0.60	12.2	19	-84954
NA	0.69	18.3	17	-67368
NC	0.69	6.6	18	-73545
NS	0.55	11.4	16	-58759
FA	0.57	18.7	9	-37227
FC	0.67	25.9	10	-37458
FS	0.51	18.6	8	-32789

Note. VA: Verbal Analogies. VC: Verbal Classification, VS: Verbal Series, NA: Numerical Analogies, NC: Numerical Classification, NS: Numerical Series, FA: Figural Analogies, FC: Figural Classification, FS: Figural Series. r_{xx}: empirical estimate of subtest reliability (EAP factor scores), % variance: proportion of variance in item-level data explained by the 2PL model, K items: number of items retained for the subtest after local misfit analysis, -LL: log-likelihood function.

Supplementary Table S3. Summary of fit of alternative latent variable / confirmatory factor analysis (CFA) models fit to Aurora data

**Model**	**Corr**	**Y-B X^2**	**df**	***p***	**CFI**	**RMSEA**	**(95% CI)**
Domain	Yes	157.257	24	<0.00001	0.976	0.029	(0.025 – 0.033)
Type	Yes	335.294	24	<0.00001	0.939	0.045	(0.042 – 0.050)
Domain+Type	No	929.061	18	<0.00001	0.677	0.123	(0.118 – 0.127)
Domain+Type	Yes	36.392	15	0.00155	0.996	0.014	(0.009 – 0.020)
2nd-order g	No	157.257	24	<0.00001	0.976	0.029	(0.025 – 0.033)
**Bifactor***	**No**	**126.415**	**18**	**<0.00001**	**0.979**	**0.031**	**(0.026 – 0.036)**
Reduced bifactor	No	157.529	21	<0.00001	0.975	0.032	(0.027 – 0.036)

Note. Corr: inclusion of estimated factor covariances. Y-B X^2: Yuan and Bentler's residual-based test statistic. CFI: Comparative Fit Index, RMSEA: Root Mean Square Error of Approximation**.** *: final model selected for the estimation of factor scores.

Supplementary Table S4. Description of ROH and CNV calls in the study sample

		Segments (per person)			Cumulative length (per person)				
		Min	*Max*	*Median*	*Min*	*Max*	*Mean*	*Median*	*SD*
**ROHs**	A	19	103	55	9,401,361	70,610,088	34,837,392	34,709,328	11,761,327
Prior to	B	0	41	8	0	91,889,339	21,693,414	18,251,557	16,318,024
CNV	C	0	33	2	0	533,575,331	61,849,913	18,030,970	84,960,332
exclusion	A+B+C	19	141	71	9,640,918	643,487,310	118,380,719	7,741,540	98,738,348
**ROHs**	A	14	84	41	6,747,526	53,417,618	25,478,418	25,128,933	8,726,857
Post	B	0	21	4	0	42,254,342	9,595,242	7,813,738	7,766,295
CNV	C	0	4	0	0	42,725,385	3,141,082	0	6,145,696
exclusion	A+B+C	14	95	46	6,747,526	90,624,411	38,214,742	35,890,473	16,736,061
**CNVs**	Gains	0	38	1	0	19,331,378	337,885	144,480	1,081,031
	Losses	0	62	1	0	11,366,652	468,561	119,794	1,070,476
	Gain+Losses	0	63	2	0	19,384,334	806,446	360,001	1,505,694

Supplementary Table S5. Nominally significant (P < 0.05) genic sCNV associations with Aurora

sCNV	*B*	*SE*	*T*	*P*	Chr	Start	End	Length	FREQ	Gene	Pheno
sLOSS_79	-16.28	6.12	-2.66	0.008405509	chr10	47,543,322	47,646,751	103,430	0.0170	*PTPN20, GLUD1P2*	Aurora
sGAIN_152	-19.47	7.47	-2.61	0.009748708	chr7	157,791,030	157,803,711	12,682	0.0113	*PTPRN2*	Aurora
sGAIN_36	10.66	4.23	2.52	0.012433463	chr16	818,802	820,215	1,414	0.0368	*MSLN, MIR662*	Aurora
sGAIN_35	11.37	4.58	2.48	0.013800989	chr16	802,234	818,801	16,568	0.0312	*MSLN*	Aurora
sGAIN_39	12.34	5.05	2.45	0.015191494	chr16	831,497	840,769	9,273	0.0255	*RPUSD1, CHTF18*	Aurora
sLOSS_79	14.46	6.14	2.35	0.019430603	chr10	47,543,322	47,646,751	103,430	0.0170	*PTPN20, GLUD1P2*	Aurora
sGAIN_48	-14.28	6.15	-2.32	0.021019379	chr10	15,045,635	15,057,374	11,740	0.0170	*ACBD7, DCLRE1C*	Aurora
sLOSS_15	16.98	7.50	2.26	0.024459566	chr21	47,821,589	47,848,458	26,870	0.0113	*PCNT*	Aurora
sLOSS_747	16.97	7.50	2.26	0.024557634	chr6	135,842,804	135,983,115	140,312	0.0113	*LINC00271*	Aurora
sGAIN_34	10.76	4.81	2.24	0.026104405	chr16	778,597	802,233	23,637	0.0283	*NARFL, HAGHL*	Aurora
sGAIN_46	-12.72	5.71	-2.23	0.026772963	chr10	14,996,416	15,027,213	30,798	0.0198	*DCLRE1C, MEIG1*	Aurora
sGAIN_52	9.78	4.41	2.22	0.027488757	chr10	135,284,115	135,343,737	59,623	0.0340	*CYP2E1, SCART1*	Aurora
sGAIN_33	11.13	5.06	2.20	0.028768331	chr16	772,842	778,596	5,755	0.0255	*CCDC78, HAGHL*	Aurora
sLOSS_279	16.17	7.51	2.15	0.032250953	chr8	144,886,809	144,940,778	53,970	0.0113	*MIR937, PUF60, SCRIB, NRBP2, EPPK1*	Aurora
sLOSS_210	13.07	6.16	2.12	0.034882803	chr14	105,163,532	105,189,504	25,973	0.0170	*INF2*	Aurora
sLOSS_429	12.93	6.16	2.10	0.036886455	chr10	27,624,562	27,703,017	78,456	0.0170	*PTCHD3*	Aurora
sGAIN_111	-13.98	6.73	-2.08	0.038905817	chr7	157,734,314	157,791,029	56,716	0.0142	*PTPRN2*	Aurora
sLOSS_747	15.50	7.51	2.06	0.04016544	chr6	135,842,804	135,983,115	140,312	0.0113	*LINC00271*	Aurora
sGAIN_39	-10.32	5.06	-2.04	0.042757981	chr16	831,497	840,769	9,273	0.0255	*RPUSD1,CHTF18*	Aurora
sGAIN_40	10.91	5.36	2.04	0.042869387	chr16	840,770	855,732	14,963	0.0227	*PRR25,GNG13,CHTF18*	Aurora
sGAIN_152	15.07	7.52	2.00	0.046146435	chr7	157,791,030	157,803,711	12,682	0.0113	*PTPRN2*	Aurora
sLOSS_16	13.40	6.74	1.99	0.047821795	chr21	47,848,459	47,856,909	8,451	0.0142	*PCNT*	Aurora

Supplementary Table S6. Linear regression parameter estimates for Class 1 (with permutation P-values)

Parameter	B	P
PC1	11.91	0.8431
PC2	-30.48	0.6667
PC3	49.67	0.9020
PC4	-34.91	0.8627
PC5	14.32	0.7451
fROH(A)	3.77	0.7647
fROH(B)	7.23	0.1600
fROH(C)	-2.21	0.0185
CNV burden	-49.57	0.0018

Supplementary Table S7. Quantile regression parameter estimates for latent class 3 (Aurora-G is the dependent variable)

Quantile	Coefficient	B	SE	T	P
25	Intercept	103.06	9.64	10.69	0
	PC1	91.16	70.84	1.29	0.200230159
	PC2	-33.70	75.81	-0.44	0.657374663
	PC3	30.25	338.94	0.09	0.929007806
	PC4	-12.13	47.10	-0.26	0.797131533
	PC5	-31.86	102.00	-0.31	0.755217515
	fROH(A)	-4.46	8.73	-0.51	0.610214211
	fROH(B)	-3.29	9.51	-0.35	0.730229045
	fROH©	-5.98	12.05	-0.50	0.620272590
	CNV burden	139.26	138.09	1.01	0.314930608
50	Intercept	110.20	7.82	14.10	0
	PC1	81.22	65.86	1.23	0.219524329
	PC2	-72.16	64.83	-1.11	0.267569514
	PC3	114.77	301.40	0.38	0.703920201
	PC4	34.86	38.98	0.89	0.372778165
	PC5	-2.30	89.14	-0.03	0.979433912
	fROH(A)	5.31	7.93	0.67	0.503766932
	fROH(B)	-15.94	9.27	-1.72	0.087888655
	fROH©	-1.93	10.87	-0.18	0.859205711
	CNV burden	17.97	118.61	0.15	0.879788332
75	Intercept	115.02	8.50	13.53	0
	PC1	-6.53	61.27	-0.11	0.915232854
	PC2	-66.23	46.82	-1.41	0.159351919
	PC3	205.05	257.82	0.80	0.427756194
	PC4	83.50	37.01	2.26	0.025606803
	PC5	64.17	92.88	0.69	0.490770728
	fROH(A)	4.51	7.91	0.57	0.569429334
	fROH(B)	-7.37	9.30	-0.79	0.429569802
	fROH©	0.62	11.88	0.05	0.958679955
	CNV burden	82.71	125.97	0.66	0.512532206

Supplementary Table S8. Quantile regression parameter estimates for latent Classes 4 and 5 (Aurora-G is the dependent variable).

Quantile	Coefficient	B	SE	T	P
25	Intercept	110.21	11.07	9.95	0
	PC1	59.60	78.35	0.76	0.448251967
	PC2	-16.53	56.46	-0.29	0.770158305
	PC3	48.12	26.06	1.85	0.067203327
	PC4	16.54	19.63	0.84	0.401085854
	PC5	-9.35	31.44	-0.30	0.766733170
	fROH(A)	-5.87	7.66	-0.77	0.445157693
	fROH(B)	5.69	3.81	1.49	0.138058727
	fROH©	-1.28	0.60	-2.11	0.036638475
	CNV burden	-63.37	114.88	-0.55	0.582200088
50	Intercept	106.11	6.43	16.51	0
	PC1	-33.96	49.83	-0.68	0.496744476
	PC2	13.66	37.38	0.37	0.715399248
	PC3	69.03	50.87	1.36	0.177141915
	PC4	2.85	18.62	0.15	0.878651807
	PC5	-16.30	26.18	-0.62	0.534598745
	fROH(A)	3.18	4.59	0.69	0.490139916
	fROH(B)	3.70	2.59	1.43	0.155695074
	fROH©	-0.14	0.42	-0.32	0.749585604
	CNV burden	-107.91	75.10	-1.44	0.153217076
75	Intercept	125.16	5.97	20.95	0
	PC1	21.88	52.76	0.41	0.679094491
	PC2	-6.61	46.38	-0.14	0.886908100
	PC3	1.47	29.51	0.05	0.960343589
	PC4	16.87	21.05	0.80	0.424322840
	PC5	-20.54	33.17	-0.62	0.536781722
	fROH(A)	-2.40	3.81	-0.63	0.529722948
	fROH(B)	1.44	2.63	0.55	0.584233969
	fROH©	-0.23	0.50	-0.45	0.653609625
	CNV burden	-214.63	74.96	-2.86	0.004906770

6 Supplementary Figures

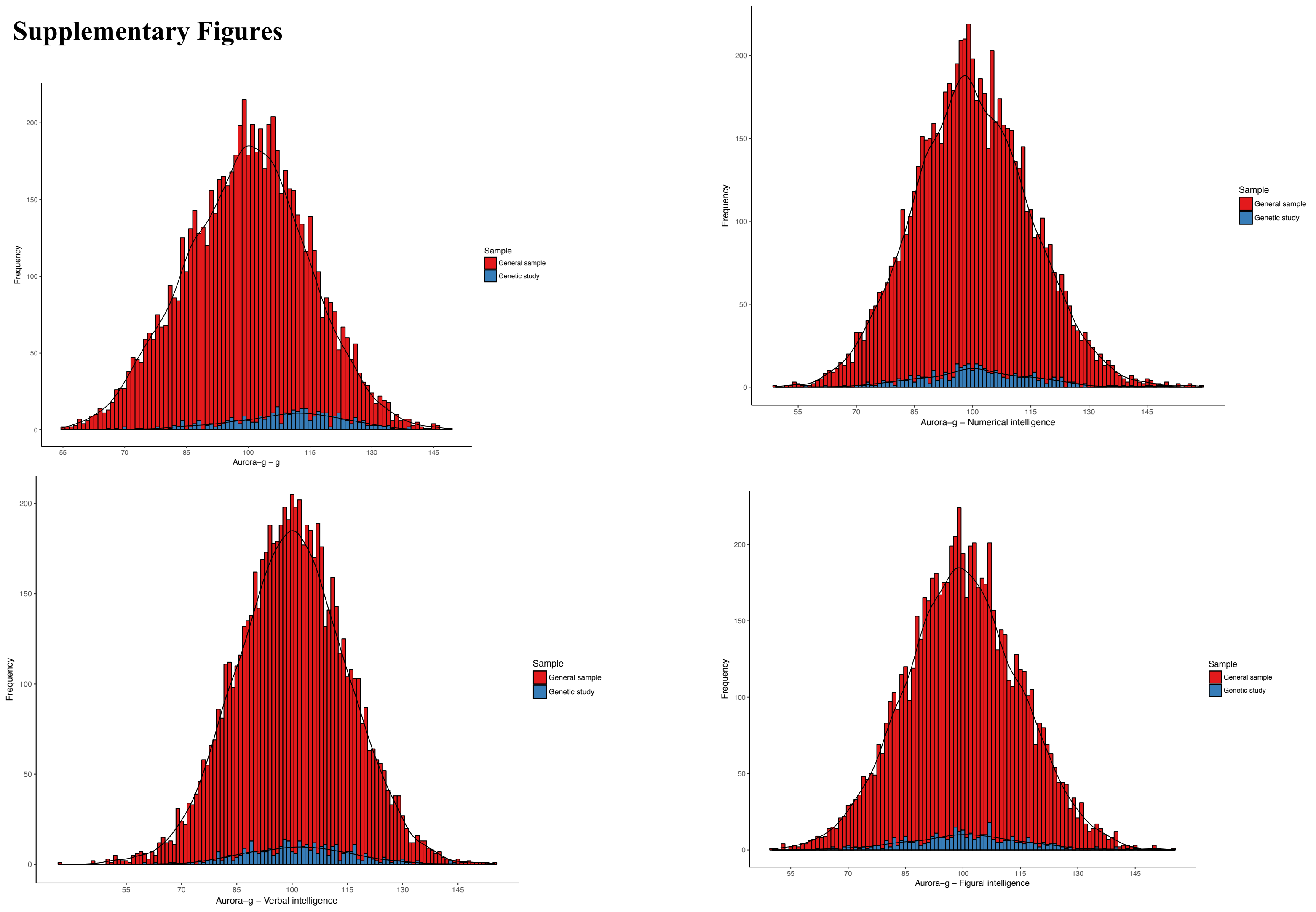

Supplementary Figure S1. Distribution of latent general cognitive ability scores in the study sample and the general population of children in Saudi Arabia

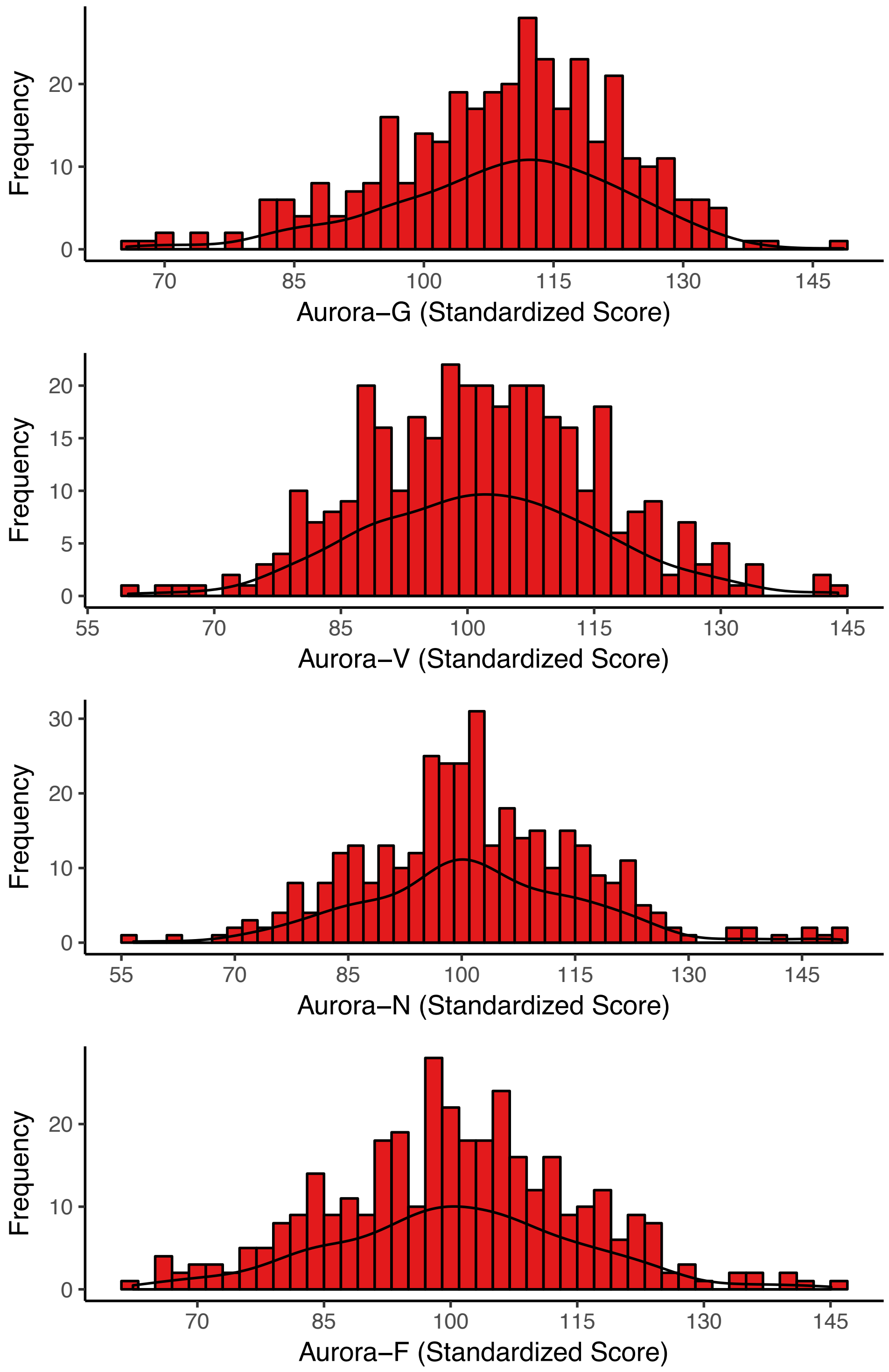

Supplementary Figure S2. Sample distributions of latent general cognitive ability estimates from Aurora. Aurora-V – verbal cognitive ability, Aurora-F: numerical cognitive ability, Aurora-S: spatial cognitive ability; Aurora-G: general cognitive ability.

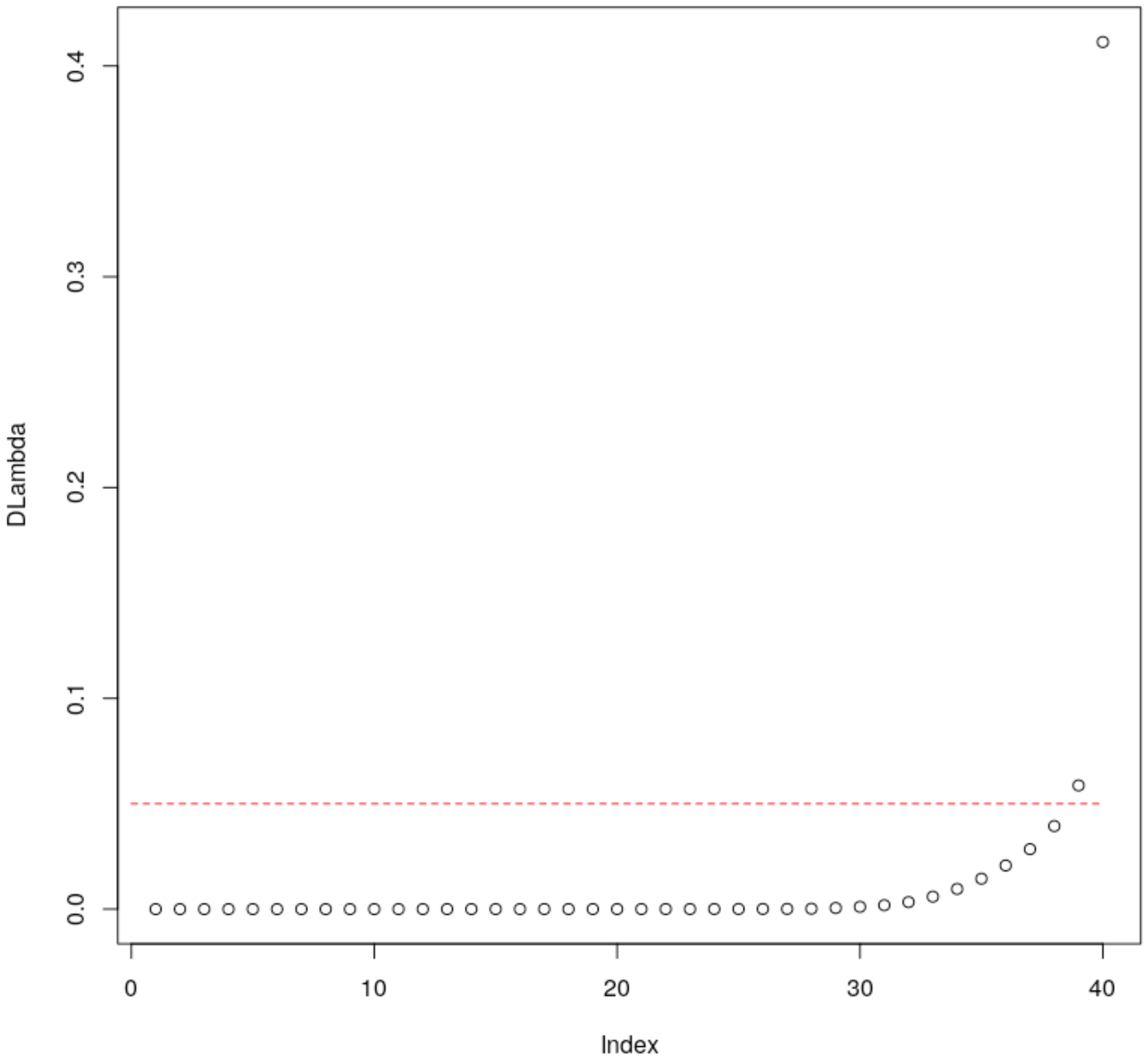

Supplementary Figure S3. Stability approach to regularization selection (StARS) tuning parameter values for CONE-based evaluation of ancestry. Red dotted line represents the default threshold parameter *β*.

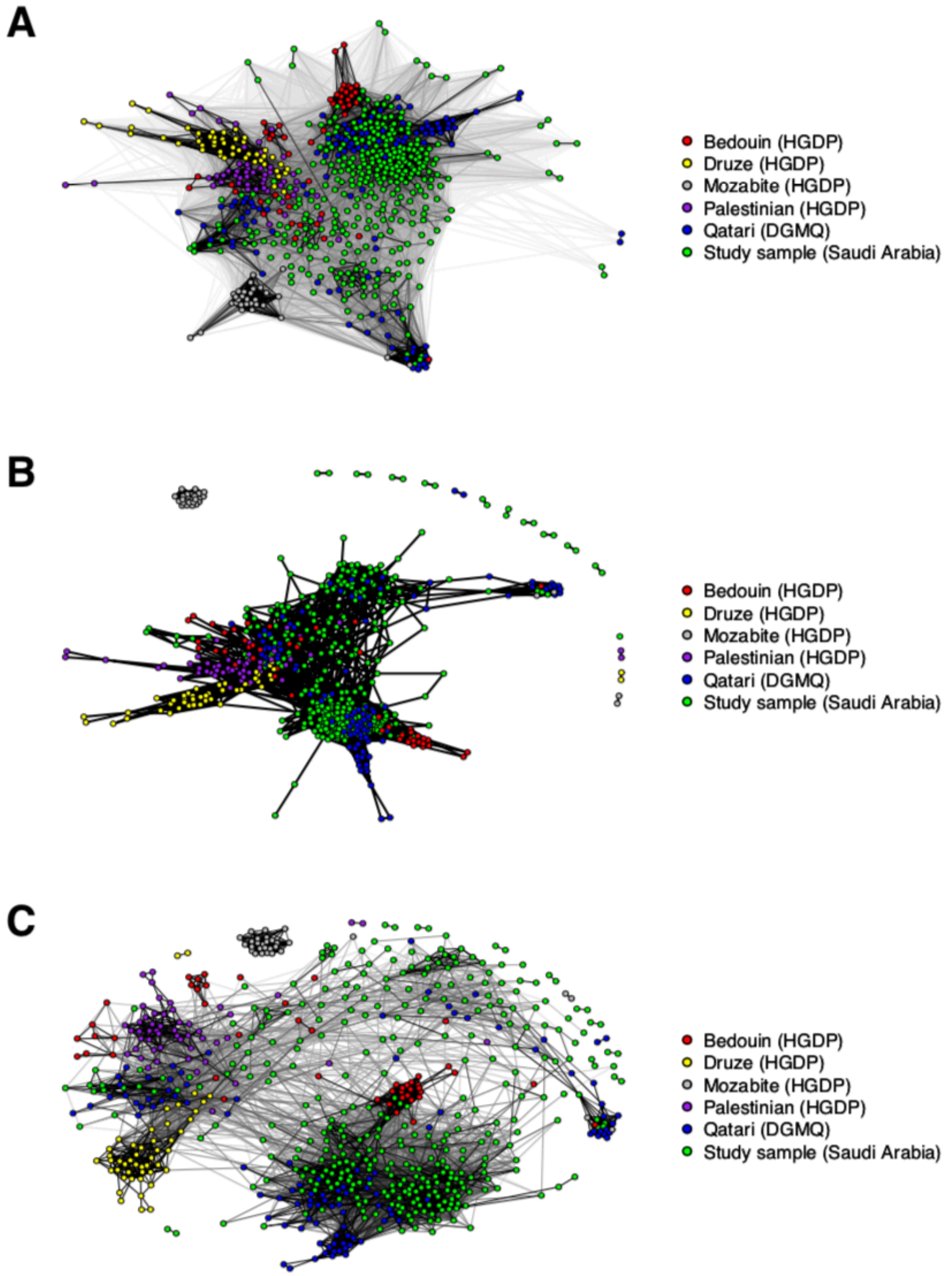

Supplementary Figure S4. Dependency graph estimated for the study sample data as well as the Middle Eastern population from HGDP and DGMQ datasets. A – StARS-based graph; B – customized neighborhood selection graph; C – combined graph.

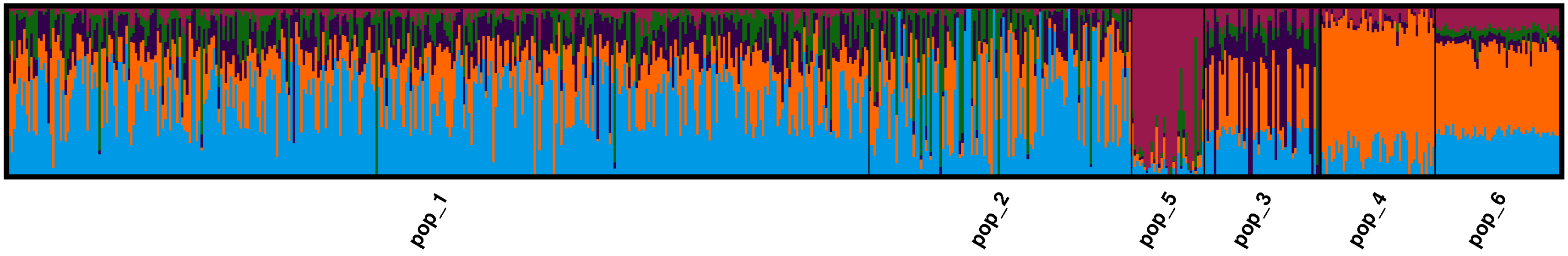

Supplementary Figure S5. Population structure graph based on ADMIXTURE analysis (for k=5 ancestral populations). Pop_1 – study sample (Saudi Arabia), pop_2 – DGMQ (Qatar) sample, pop_3 – Bedouin (HGDP), pop_4 – Druze (HGDP), pop_5 – Mozabite (HGDP), pop_6 – Palestinian (HGDP).

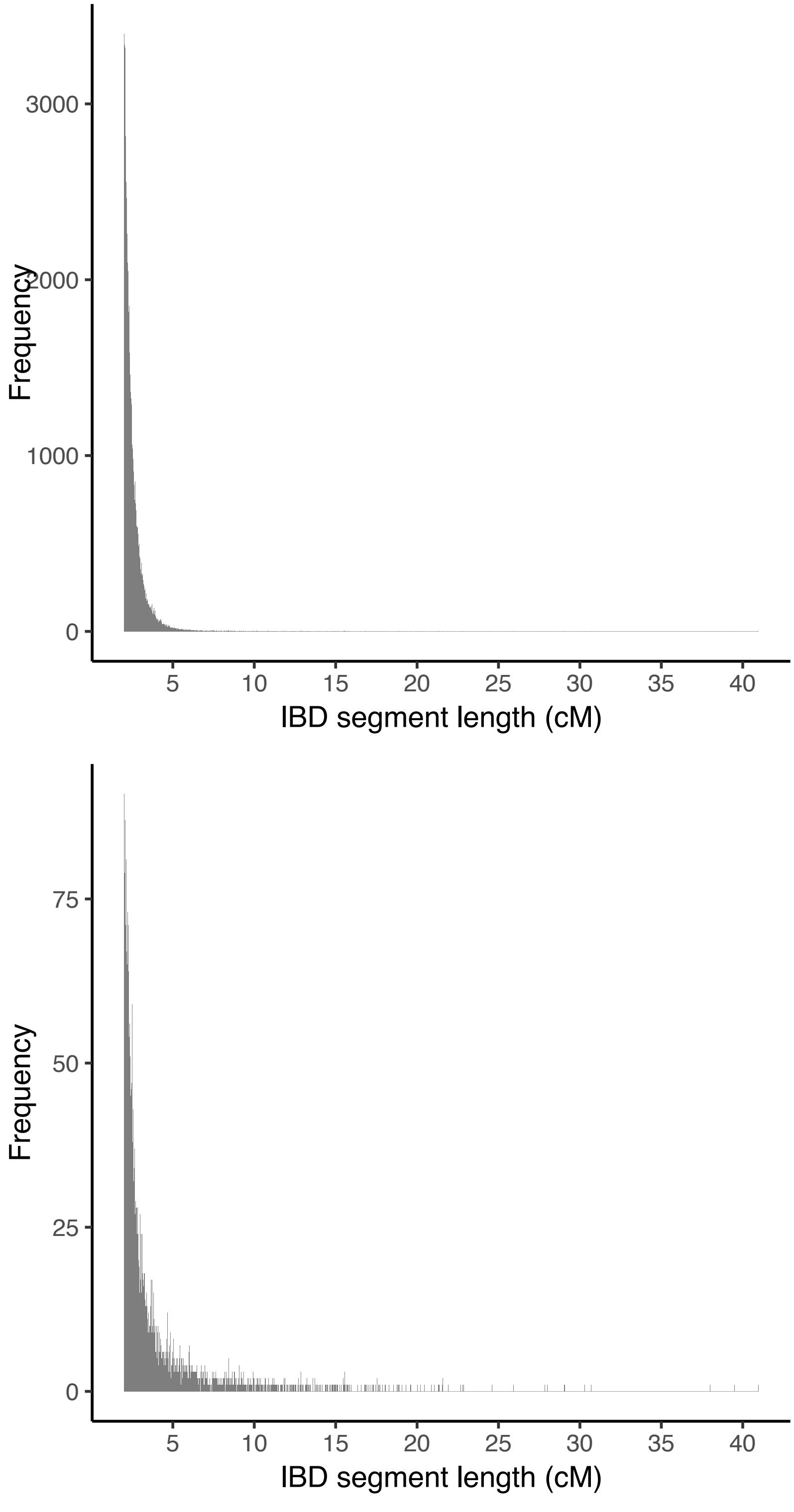

Supplementary Figure S6. IBD segment length distributions in the total sample (top panel) vs related individuals (bottom panel; up to 7th degree).

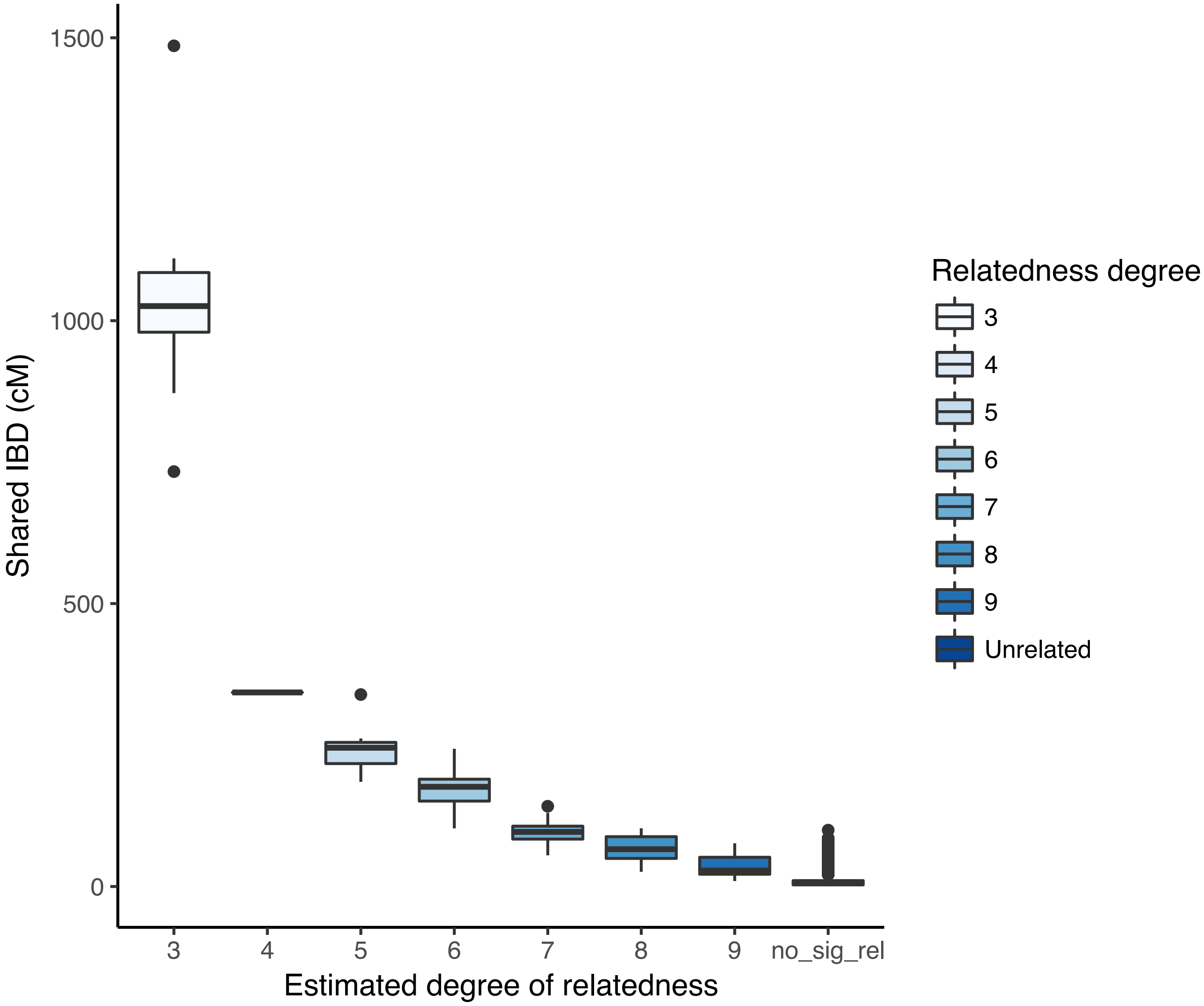

Supplementary Figure S7. Boxplots of cumulative IBD sharing among pairs of individuals of different degrees of relatedness as estimated by ERSA and FISHR2**.**

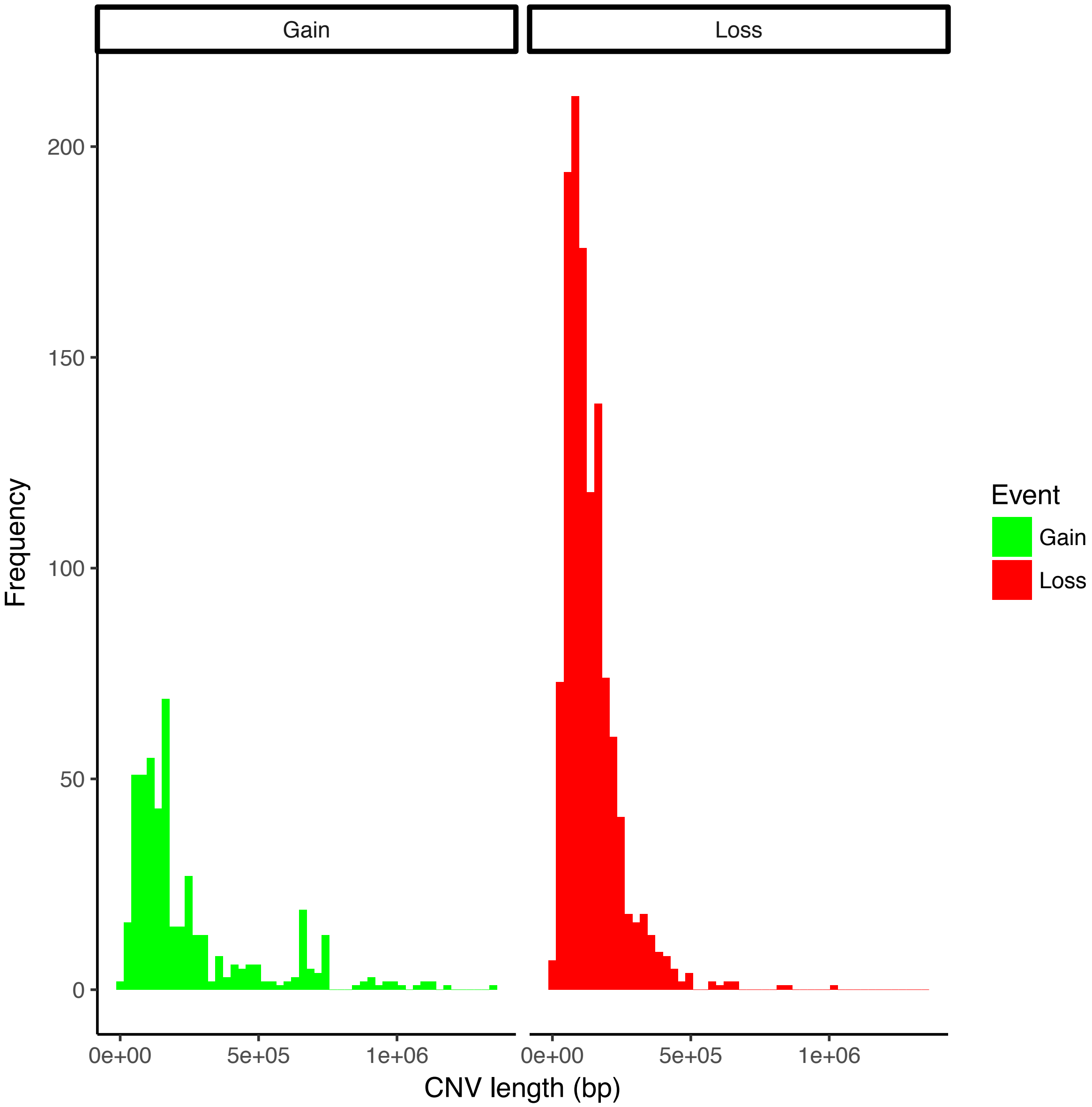

Supplementary Figure S8. CNV segment length distributions in the study sample (n=353)

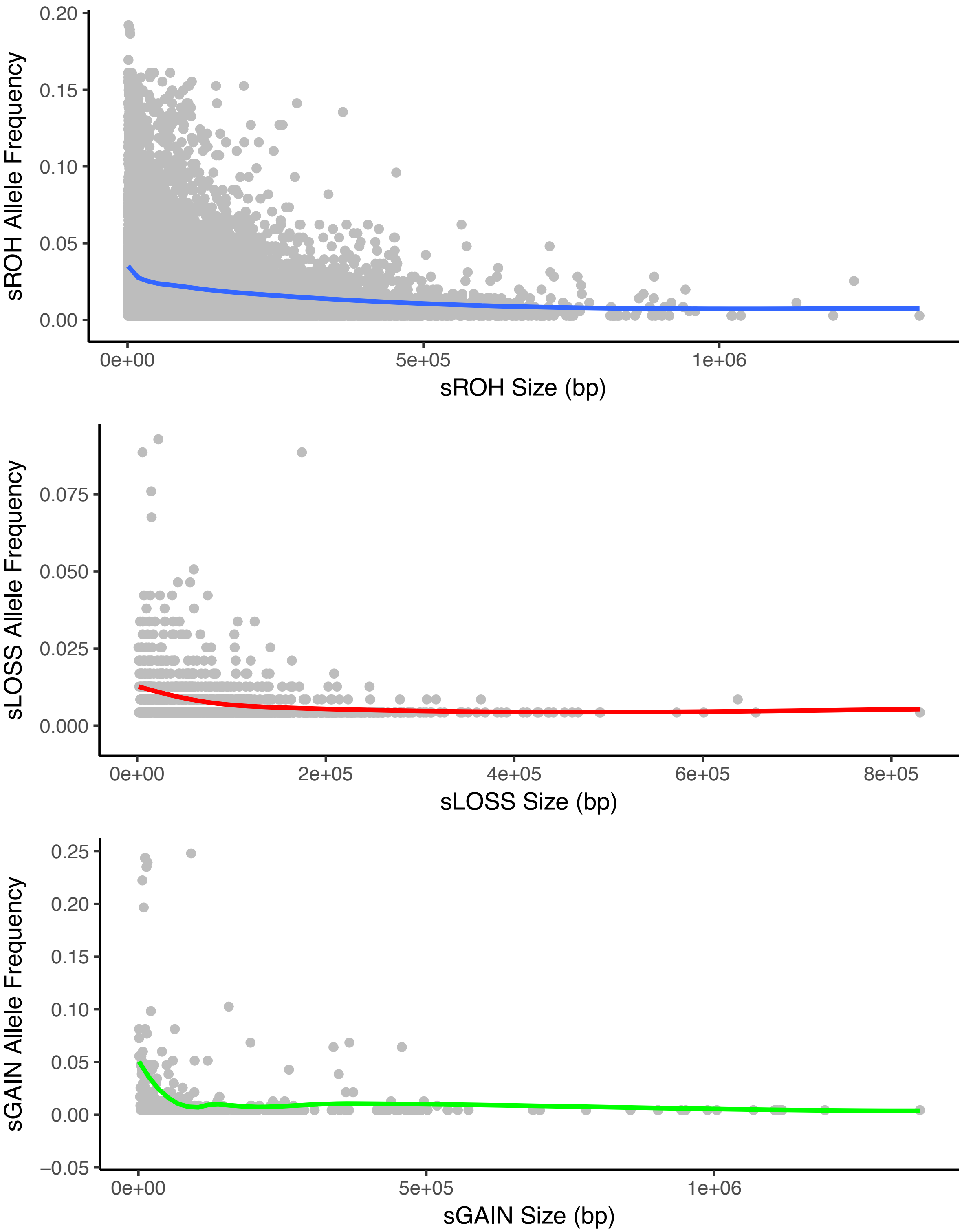

Supplementary Figure S9. Scatterplots of sROH and sCNV (sLOSS+sGAIN) allele frequencies vs. sROH and sCNV segment length

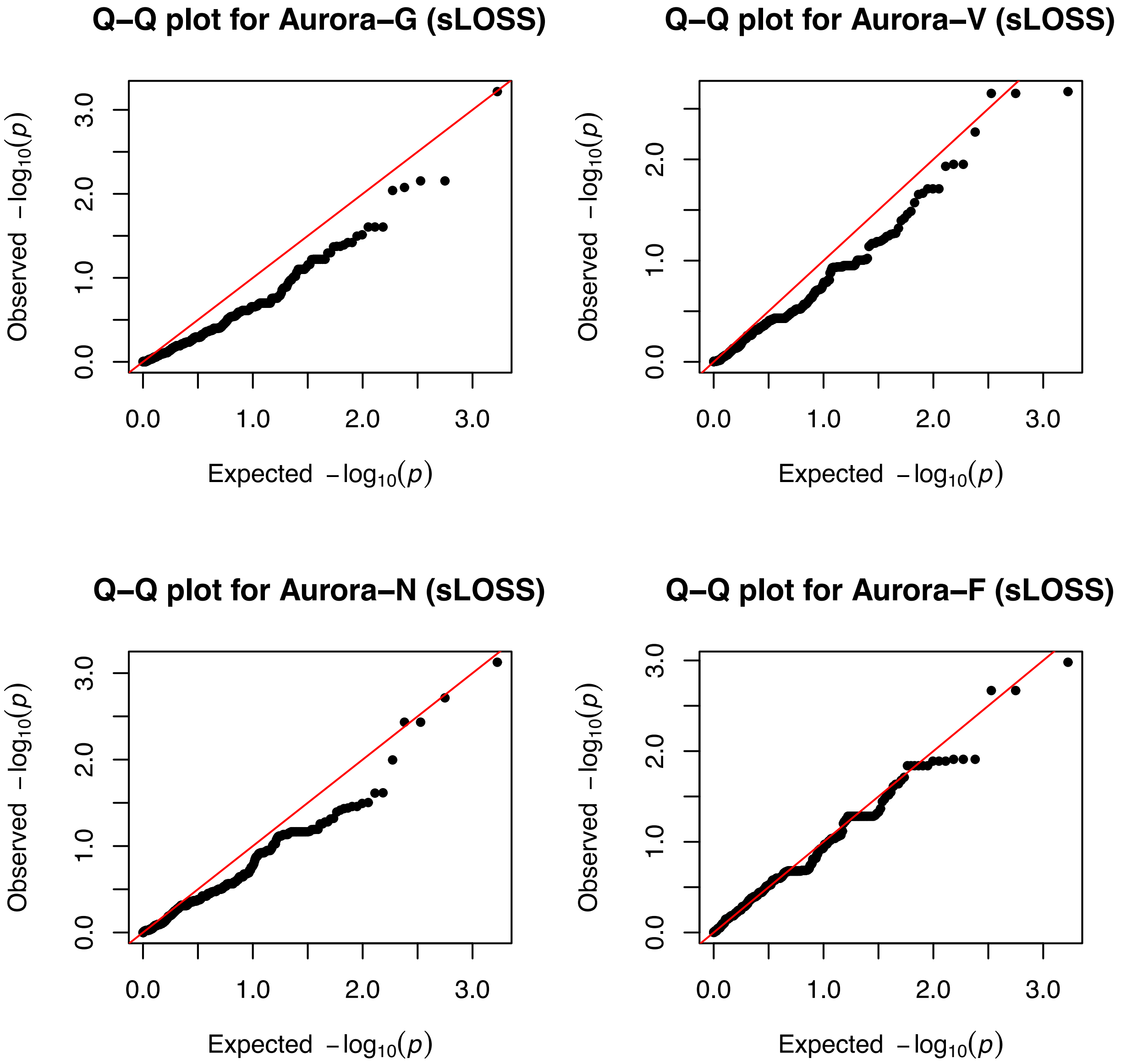
Q–Q plot for Aurora–G (sLOSS)
Q–Q plot for Aurora–V (sLOSS)
Q–Q plot for Aurora–N (sLOSS)
Q–Q plot for Aurora–F (sLOSS)
Observed −log10(p)
Expected −log10(p)
0.0
1.0
2.0
3.0

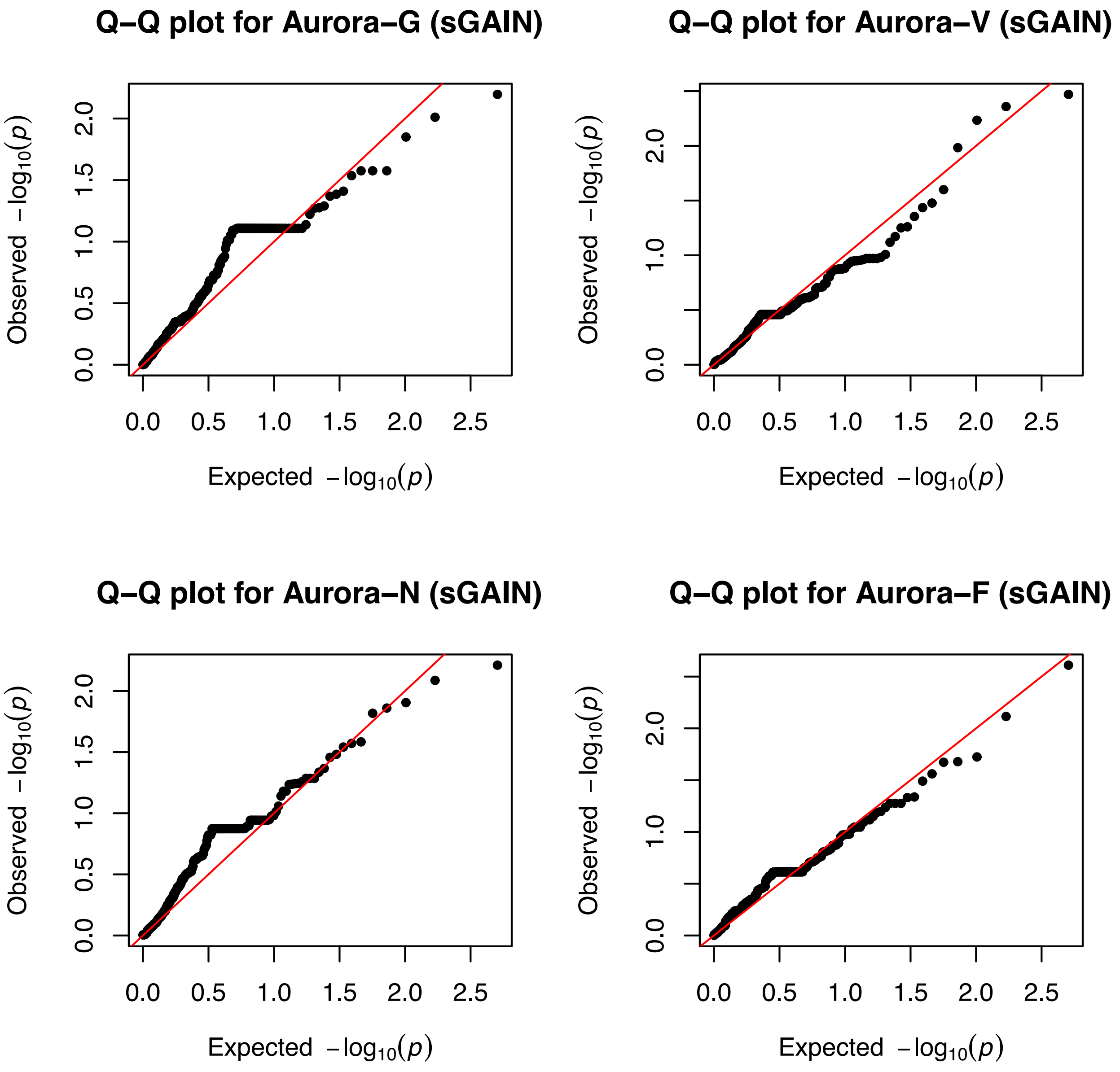

Supplementary Figure S10. Q-Q plots for sCNV-based association analysis of Aurora-g

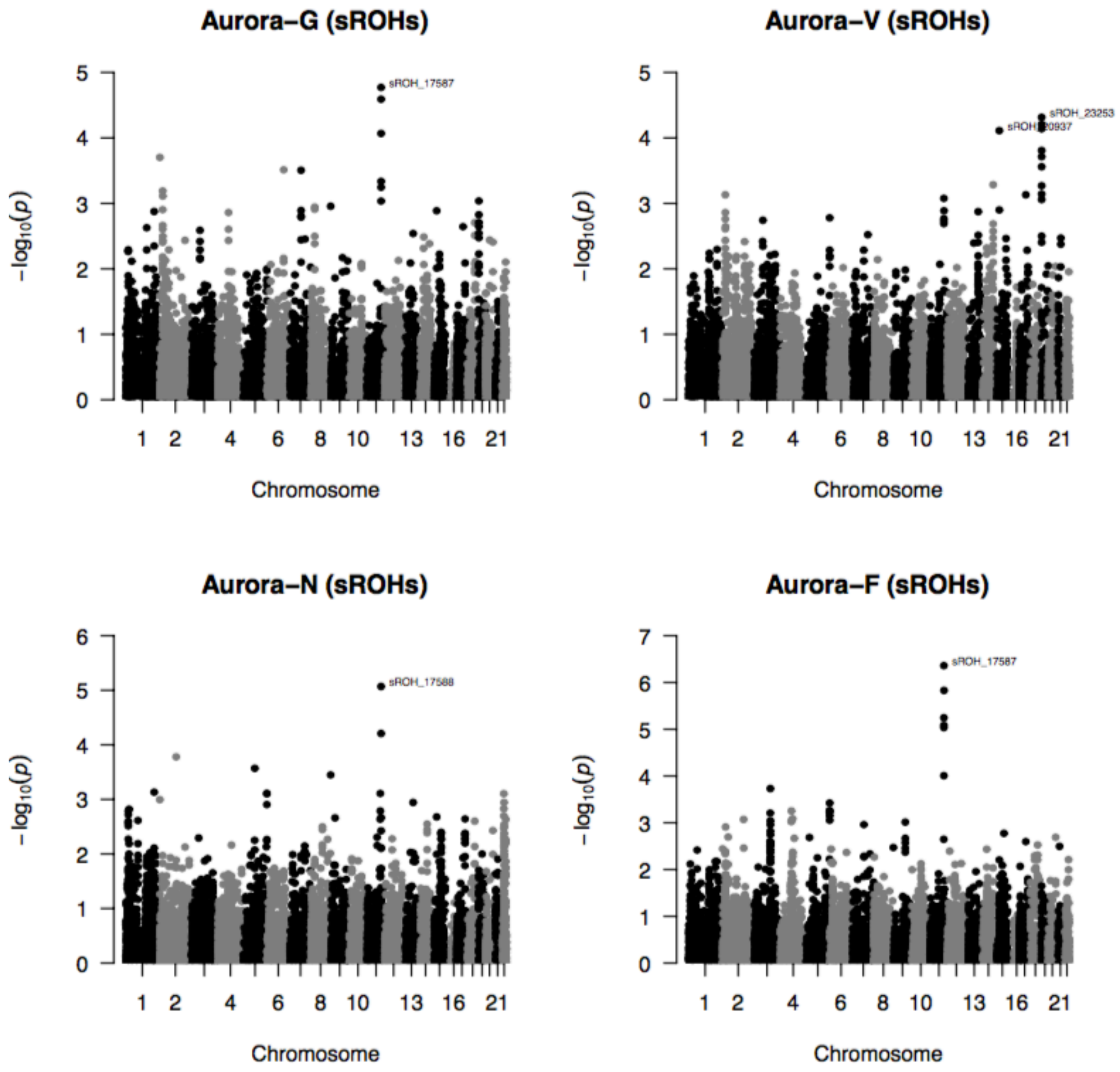

Supplementary Figure S11. Manhattan plots for sROH-based association analysis of Aurora

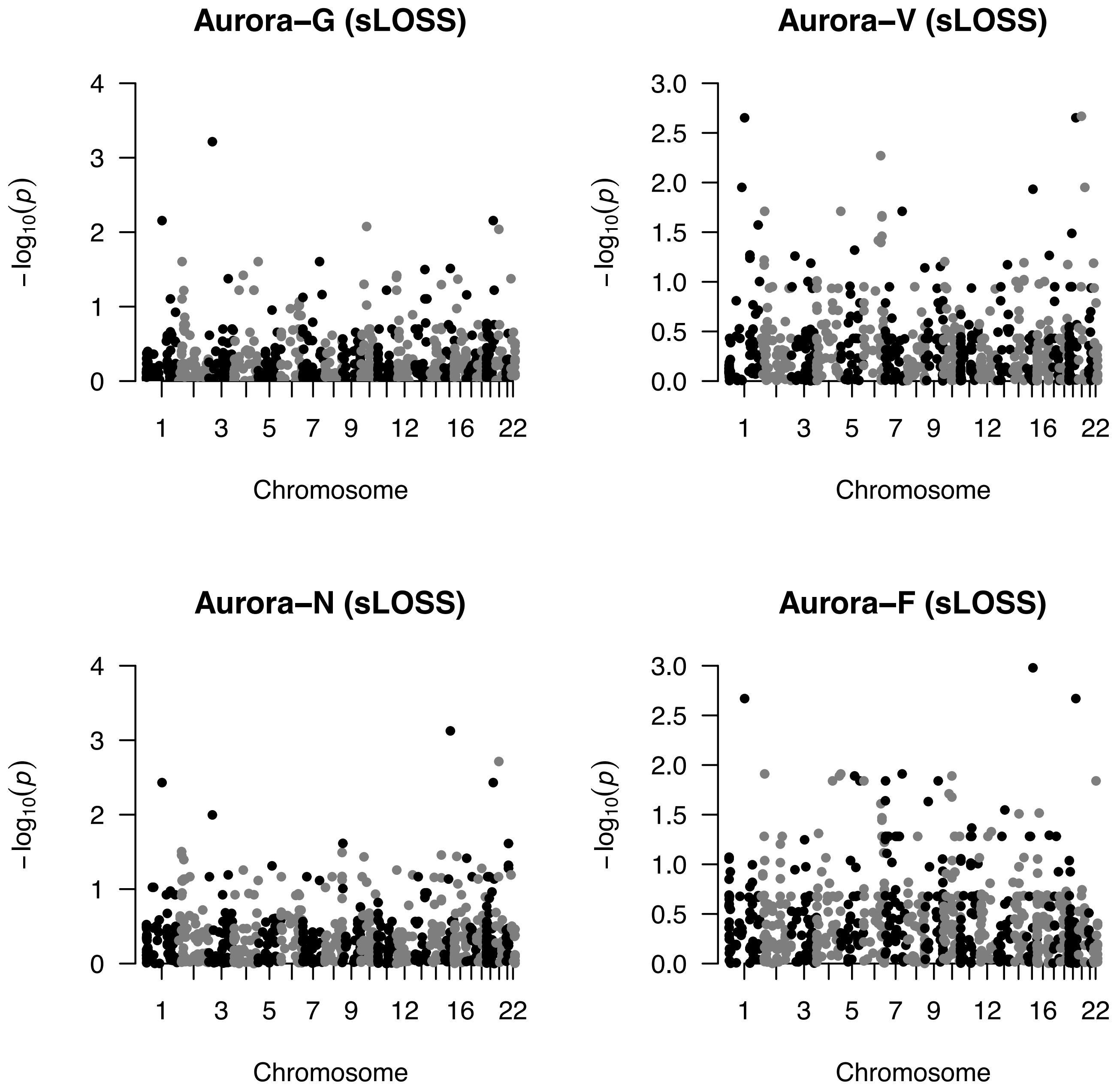
Aurora–G (sLOSS)
Aurora–V (sLOSS)
Aurora–N (sLOSS)
Aurora–F (sLOSS)
$-\log_{10}(p)$
Chromosome
0
1
2
3
4
0.0
0.5
1.0
1.5
2.0
2.5
3.0
1
3
5
7
9
12
16
22

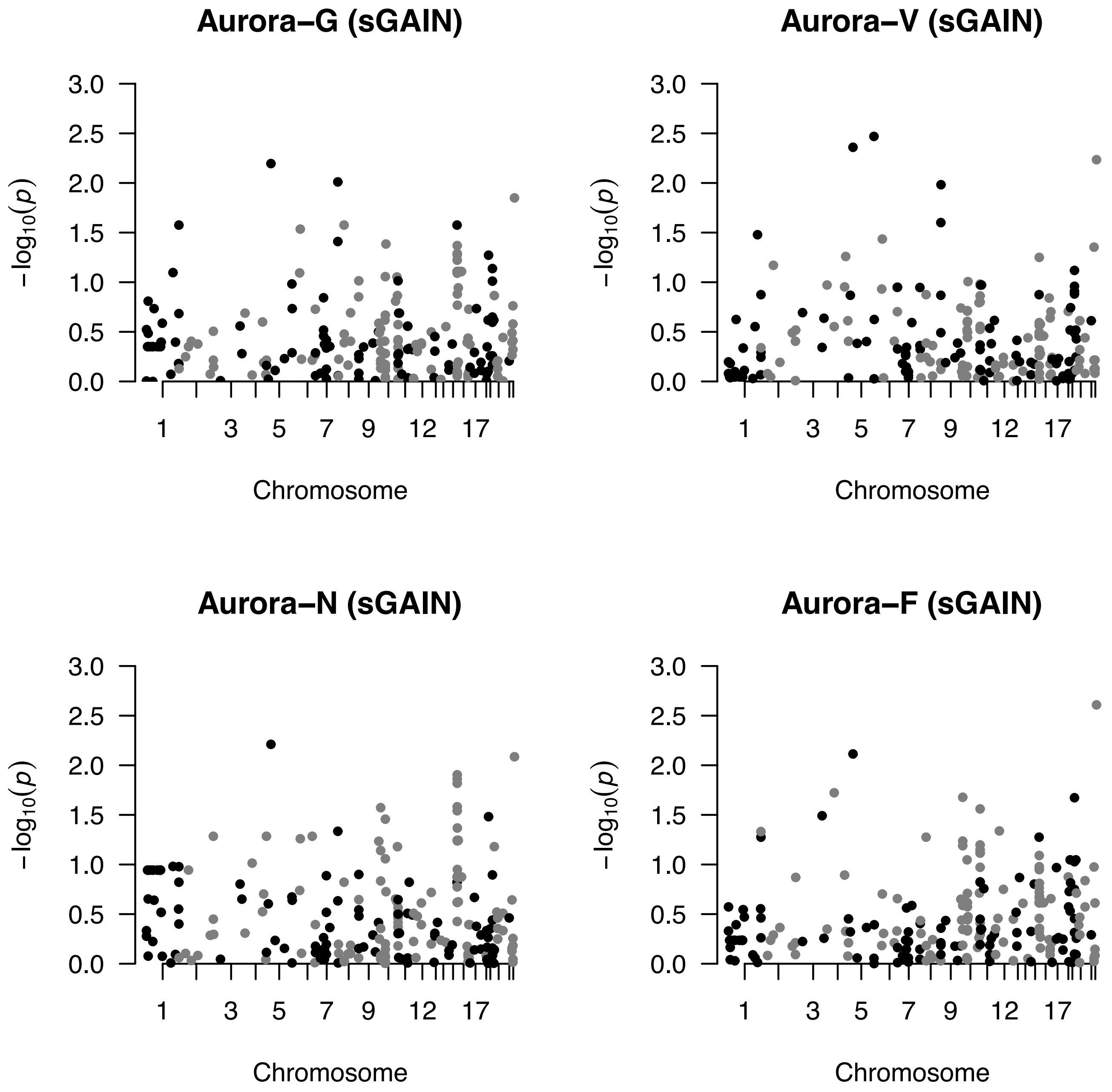

Supplementary Figure S12. Manhattan plots for sCNV-based association analysis of Aurora

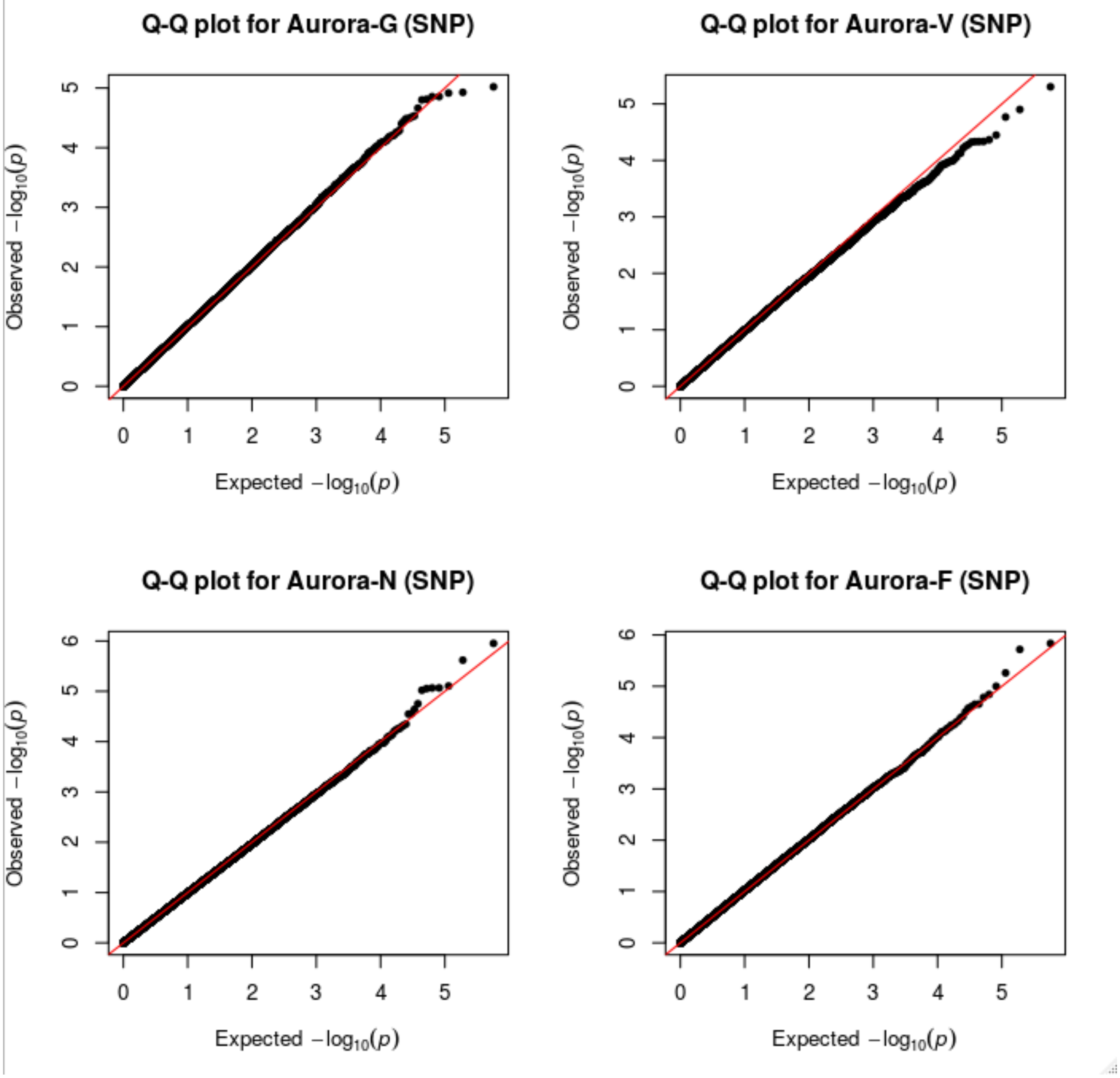

Supplementary Figure S13. Q-Q plots for SNP-based association analysis of Aurora

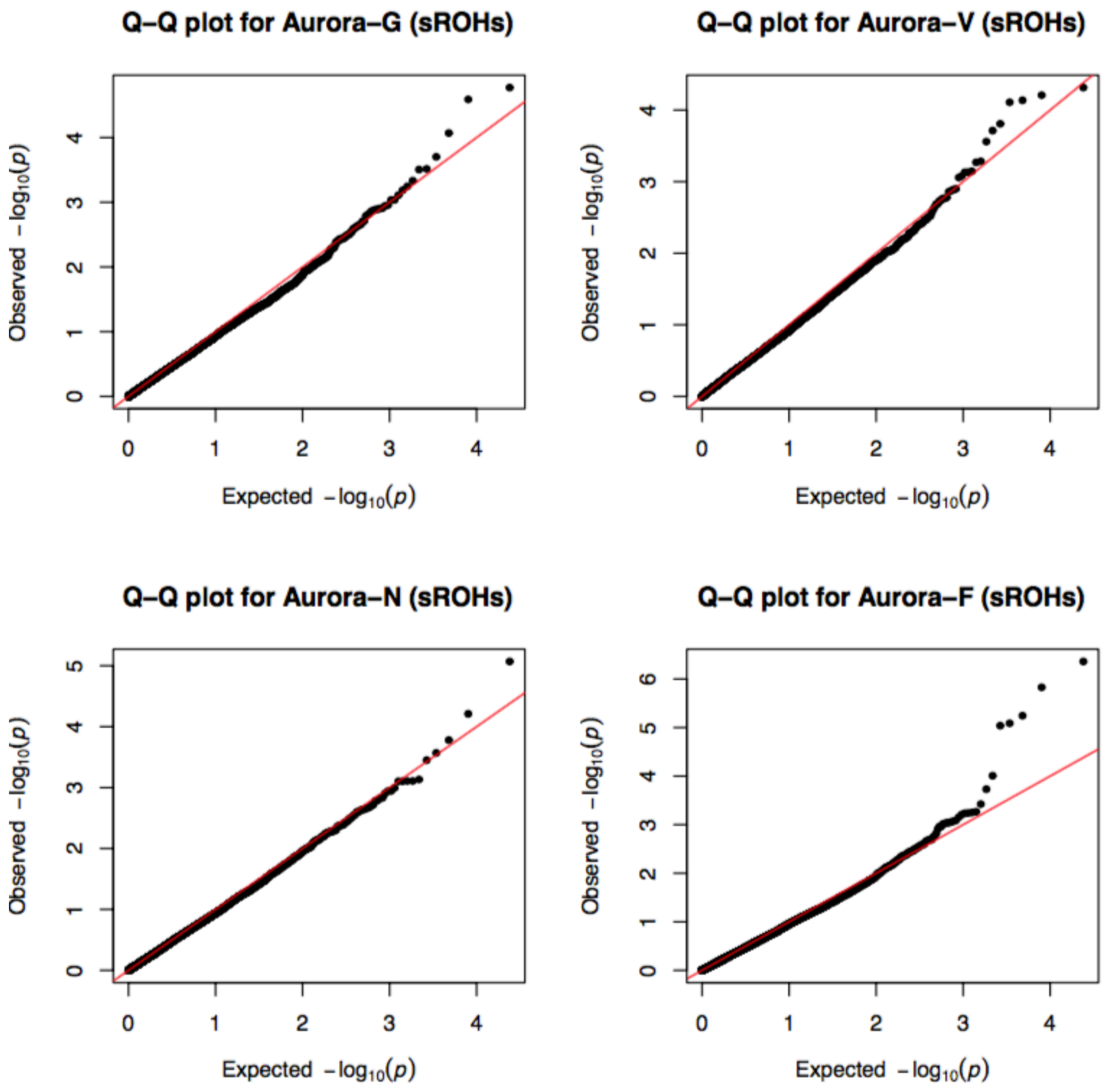

Supplementary Figure S14. Q-Q plots for sROH-based association analysis of Aurora

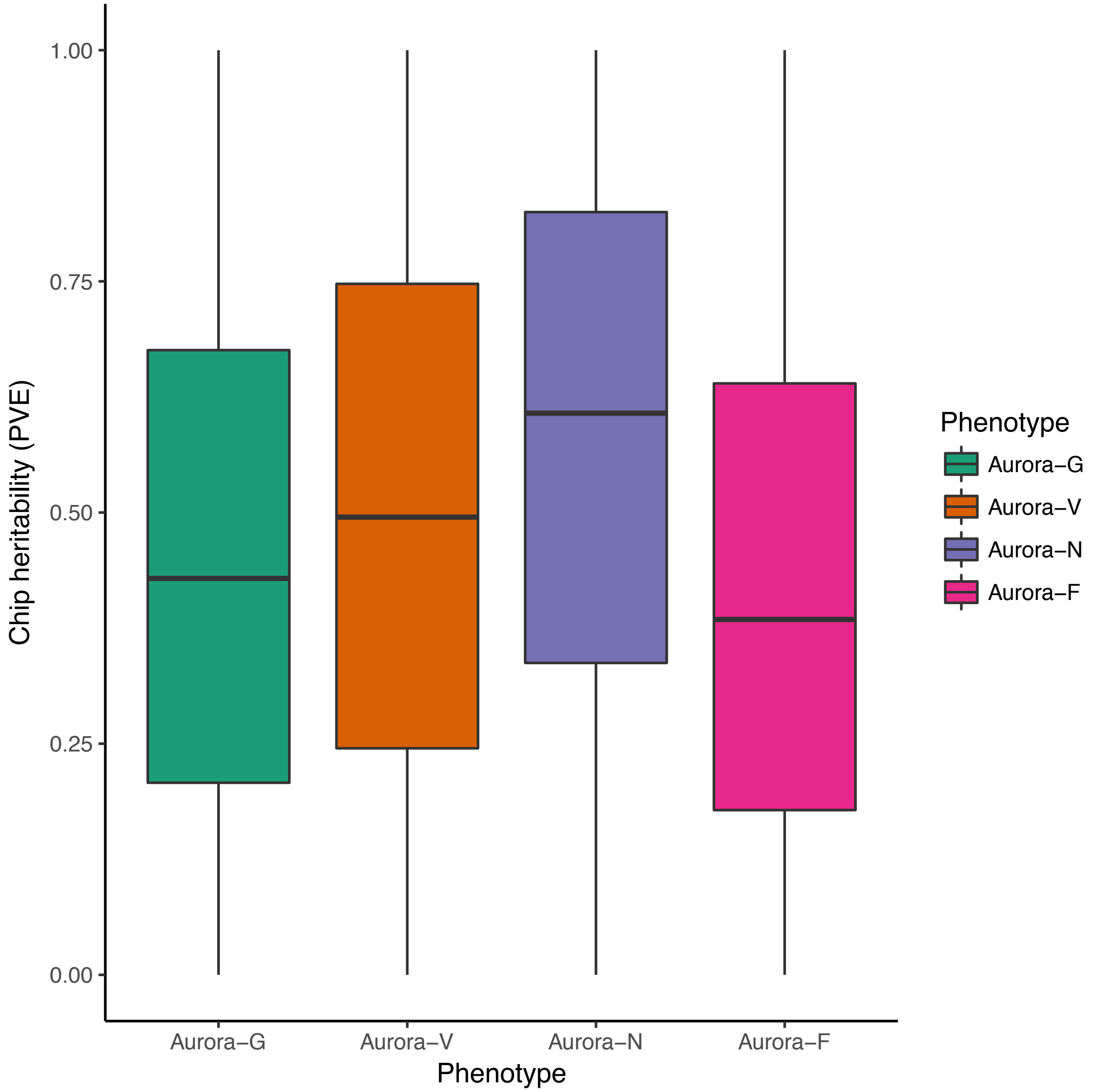

Supplementary Figure S15. Boxplots of chip heritability (PVE) estimates from GEMMA for Aurora phenotypes.

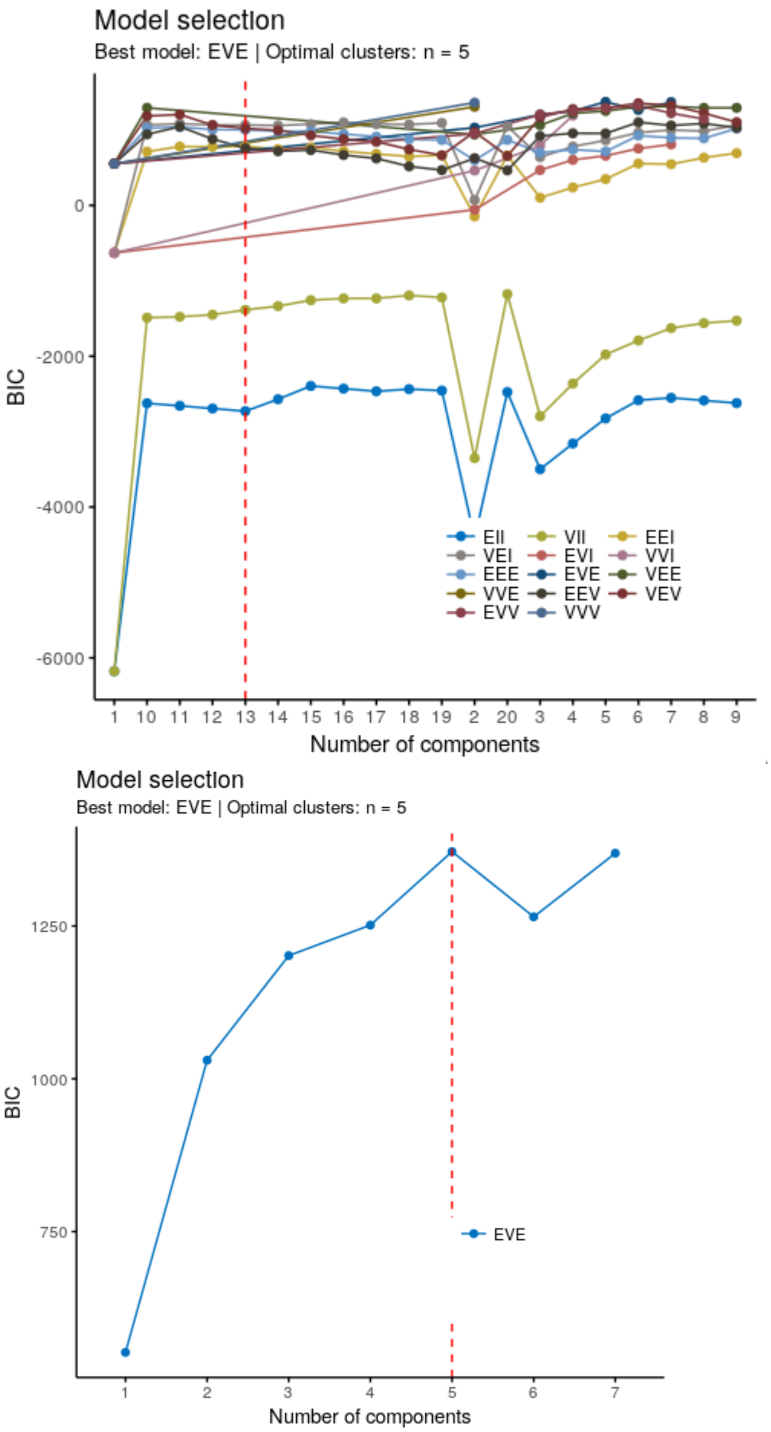

Supplementary Figure S16. Bayesian Information Criterion (BIC) values for the range of latent class models estimated using mclust

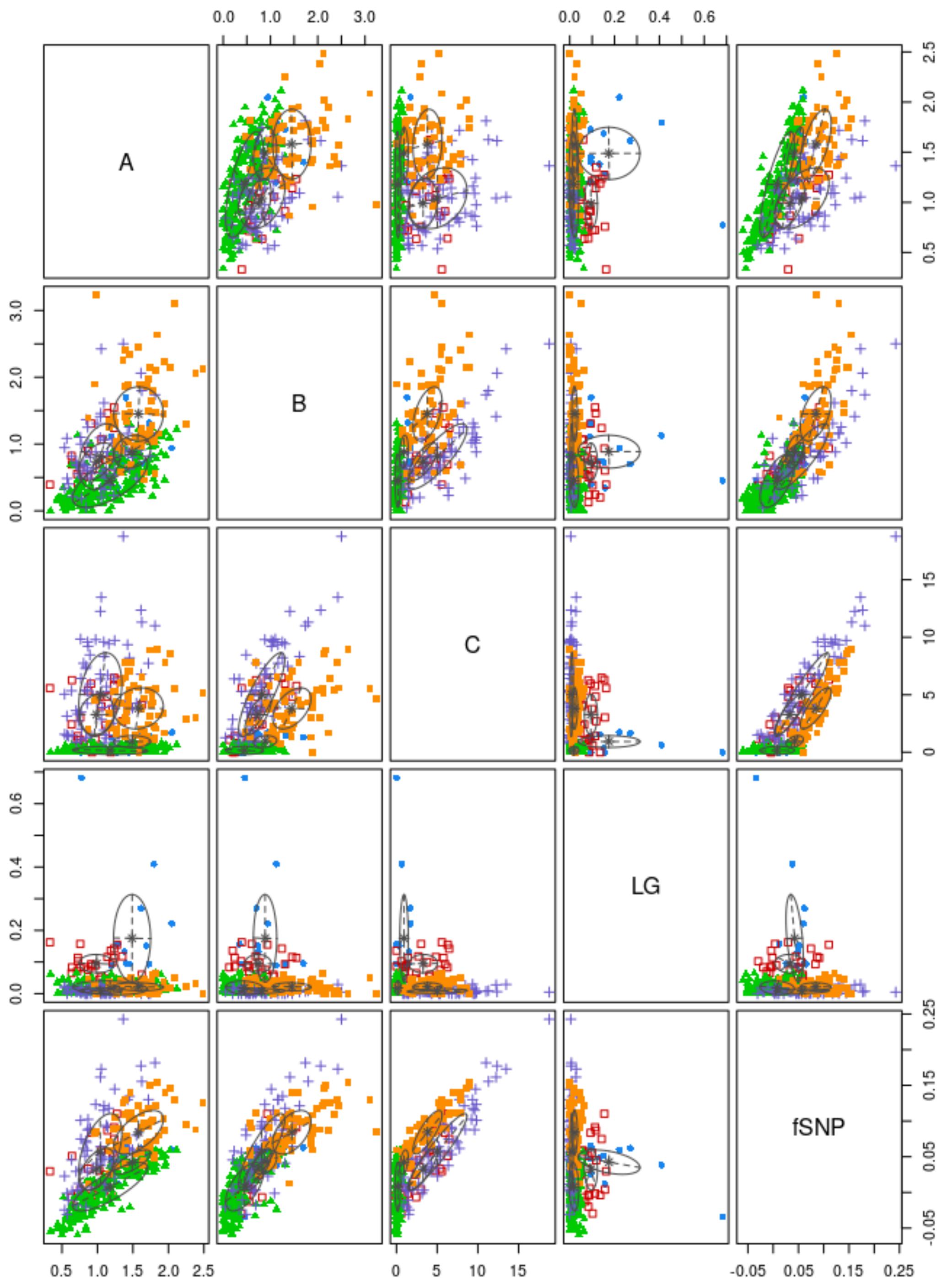

Supplementary Figure S17. Classification plot for the results of latent class analysis of homozygosity and CNV burden in the study sample. Colors represent class membership. A: fROH(A), B: fROH(B), C: fROH(C), LG: CNV burden (loss+gain).

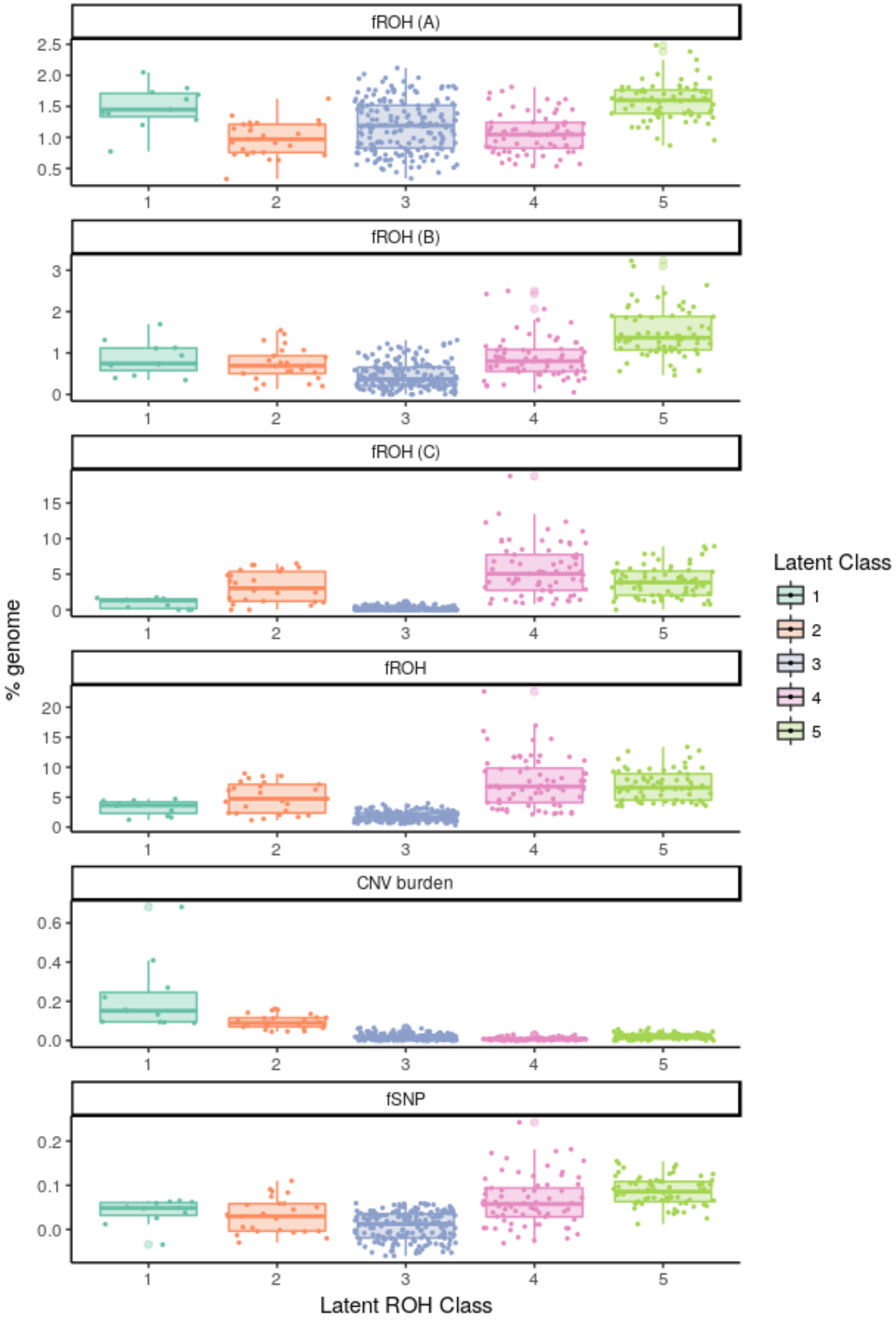

Supplementary Figure S18. Distributions of key ROH- and CNV-associated variables among five latent classes of individuals

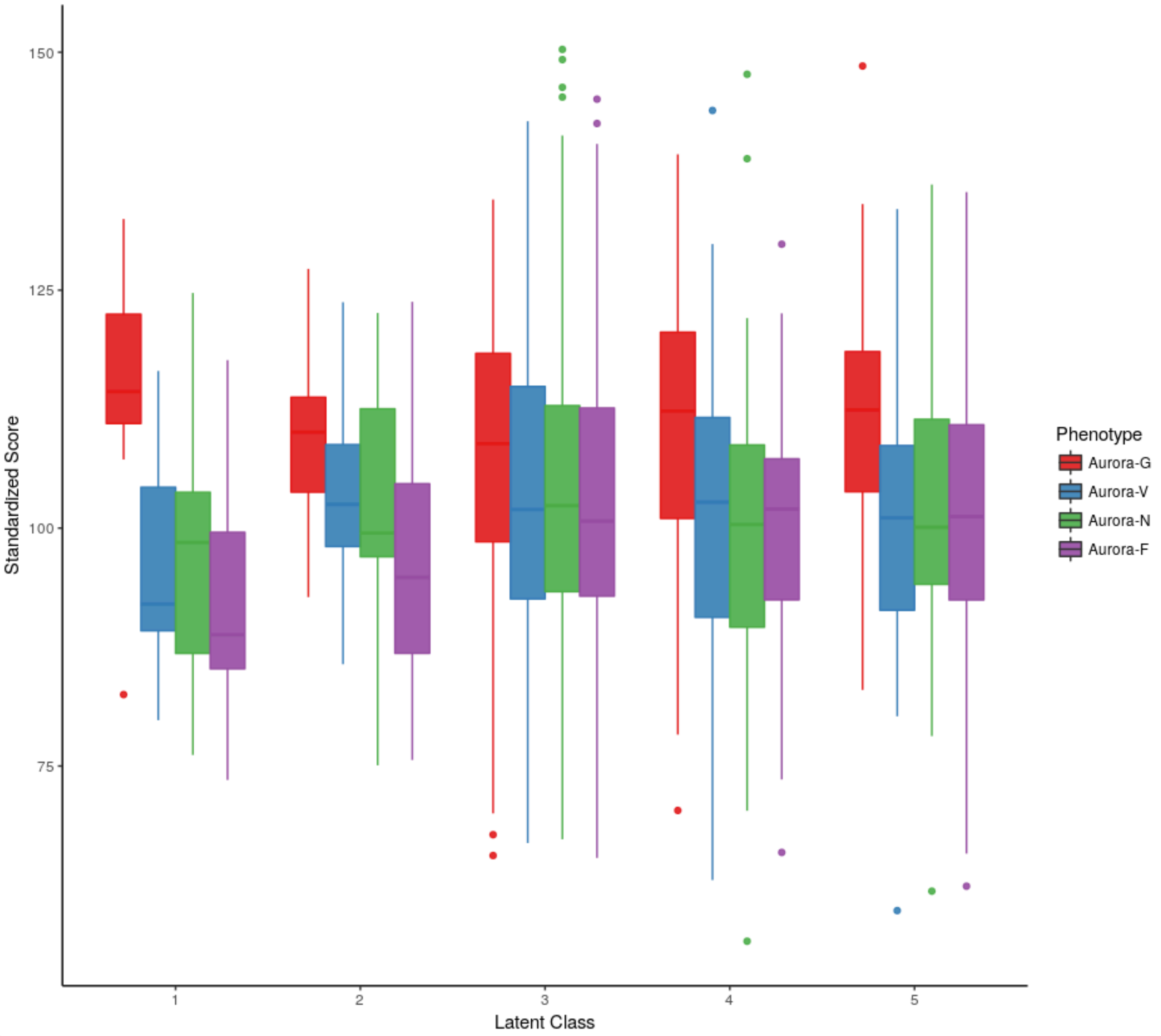

Supplementary Figure S19. Distributions of latent general cognitive ability estimates in five latent clusters of individuals

7 References

Alexander, D.H., Novembre, J., and Lange, K. (2009). Fast model-based estimation of ancestry in unrelated individuals. *Genome Research* 19**,** 1655-1664.

Bjelland, D.W., Lingala, U., Patel, P.S., Jones, M., and Keller, M.C. (2017). A fast and accurate method for detection of IBD shared haplotypes in genome-wide SNP data. *European Journal of Human Genetics* 25**,** 617-624.

Browning, B.L., and Browning, S.R. (2009). A Unified Approach to Genotype Imputation and Haplotype-Phase Inference for Large Data Sets of Trios and Unrelated Individuals. *American Journal of Human Genetics* 84**,** 210-223.

Cann, H.M., De Toma, C., Cazes, L., Legrand, M.F., Morel, V., Piouffre, L., Bodmer, J., Bodmer, W.F., Bonne-Tamir, B., Cambon-Thomsen, A., Chen, Z., Chu, J.Y., Carcassi, C., Contu, L., Du, R.F., Excoffier, L., Ferrara, G.B., Friedlaender, J.S., Groot, H., Gurwitz, D., Jenkins, T., Herrera, R.J., Huang, X.Y., Kidd, J., Kidd, K.K., Langaney, A., Lin, A.A., Mehdi, S.Q., Parham, P., Piazza, A., Pistillo, M.P., Qian, Y.P., Shu, Q.F., Xu, J.J., Zhu, S., Weber, J.L., Greely, H.T., Feldman, M.W., Thomas, G., Dausset, J., and Cavalli-Sforza, L.L. (2002). A human genome diversity cell line panel. *Science* 296**,** 261-262.

Deelen, P., Bonder, M.J., Van Der Velde, K.J., Westra, H.-J., Winder, E., Hendriksen, D., Franke, L., and Swertz, M.A. (2014). Genotype harmonizer: automatic strand alignment and format conversion for genotype data integration. *BMC Research Notes* 7**,** 901.

Gusev, A., Lowe, J.K., Stoffel, M., Daly, M.J., Altshuler, D., Breslow, J.L., Friedman, J.M., and Pe'er, I. (2009). Whole population, genome-wide mapping of hidden relatedness. *Genome Research* 19**,** 318-326.

Huff, C.D., Witherspoon, D.J., Simonson, T.S., Xing, J.C., Watkins, W.S., Zhang, Y.H., Tuohy, T.M., Neklason, D.W., Burt, R.W., Guthery, S.L., Woodward, S.R., and Jorde, L.B. (2011). Maximum-likelihood estimation of recent shared ancestry (ERSA). *Genome Research* 21**,** 768-774.

Kopelman, N.M., Mayzel, J., Jakobsson, M., Rosenberg, N.A., and Mayrose, I. (2015). Clumpak: a program for identifying clustering modes and packaging population structure inferences across K. *Mol Ecol Resour* 15**,** 1179-1191.

Kuismin, M.O., Ahlinder, J., and Sillanpää, M.J. (2017). CONE: Community Oriented Network Estimation Is a Versatile Framework for Inferring Population Structure in Large-Scale Sequencing Data. *G3: Genes|Genomes|Genetics* 7**,** 3359-3377.

Li, Y.L., and Liu, J.X. (2018). STRUCTURESELECTOR: A web-based software to select and visualize the optimal number of clusters using multiple methods. *Molecular Ecology Resources* 18**,** 176-177.

Liu, H.J., Roeder, K., and Wasserman, D. (2010). Stability Approach to Regularization Selection (Stars) for High Dimensional Graphical Models. *Advances in Neural Information Processing Systems* 23**,** 1432-1440.

Matise, T.C., Chen, F., Chen, W.W., De La Vega, F.M., Hansen, M., He, C.S., Hyland, F.C.L., Kennedy, G.C., Kong, X.Y., Murray, S.S., Ziegle, J.S., Stewart, W.C.L., and Buyske, S. (2007). A second-generation combined linkage-physical map of the human genome. *Genome Research* 17**,** 1783-1786.

Rodriguez-Flores, J.L., Fakhro, K., Agosto-Perez, F., Ramstetter, M.D., Arbiza, L., Vincent, T.L., Robay, A., Malek, J.A., Suhre, K., Chouchane, L., Badii, R., Al-Marri, A.a.N., Khalil, C.A., Zirie, M., Jayyousi, A., Salit, J., Keinan, A., Clark, A.G., Crystal, R.G., and Mezey, J.G. (2016). Indigenous Arabs are descendants of the earliest split from ancient Eurasian populations. *Genome Research* 26**,** 151-162.

Schwender, H., and Ickstadt, K. (2008). "Imputing missing genotypes with weighted k nearest neighbors", in: *Sonderforschungsbereich 475, Komplexitätsreduktion in Multivariaten Datenstrukturen.* (Dortmund: Universität Dortmund).
